# Engineering injectable bone/bioadhesive grafts delivery system with self-healing properties for bone regeneration

**DOI:** 10.1016/j.bioactmat.2025.07.049

**Published:** 2025-08-11

**Authors:** Qingzu Liu, Bin Zhu, Huikai Yang, Chongyang Liu, Yurong Chen, Xiaoyu Wu, Wangling Duan, Luyao Feng, Binhui Wang, Liang Shao, Jianpeng Gao, Yazhong Bu, Hongjian Liu, Keya Mao, Jianheng Liu

**Affiliations:** a Department of Orthopedics, The Fourth Medical Centre of Chinese PLA General Hospital, Beijing, 100089, China; b Medical School of Chinese PLA, Beijing, 100853, China; c Department of Burn/Plastic Surgery and Wound Repair, The Second Affiliated Hospital of Xi'an Jiaotong University, Xi'an, Shaanxi, 710061, China; d Institute of Medical Engineering, Department of Biophysics, School of Basic Medical Sciences, Health Science Center, Xi'an Jiaotong University, Xi'an, Shaanxi, 710061, China; e School of Medicine, Nankai University, Tianjin, 300071, China; f Department of Anesthesiology, The First Medical Center of Chinese PLA General Hospital, Beijing, 100853, China; g College of Biological Science and Engineering, Fuzhou University, Fuzhou, Fujian, 350108, China; h Department of Orthopedics, The First Affiliated Hospital of Zhengzhou University, Zhengzhou, Henan, 450052, China

**Keywords:** Bioadhesive, Bone graft, Self-healing, Hemostatic, Deliver additives

## Abstract

The incidence of open bone defects caused by high kinetic and potential energy injuries has significantly increased. Bone grafting, typically in the form of granules, is widely recognized as the most effective treatment. However, current bone graft system is not considered ideal due to issues such as mismatched shapes and dislocation. Additionally, bone defects are frequently associated with substantial bleeding, and bone graft system often fail to effectively seal and prevent leakage, increasing the post-operative complications. In this study, based on PEG active ester (Bi-PEG-SG) and gelatin, we developed a micro-scale calf bone granules/PEG-Gelatin bioadhesive grafts delivery system with self-healing properties, which not only possesses antioxidant properties but also demonstrates injectability, shape adaptability, adhesive capabilities and high bursting pressure. This system effectively addresses the displacement issues of bone grafts and shows significant sealing and hemostatic capabilities in models of femoral artery transection hemorrhage and rabbit femoral condyle bleeding. Furthermore, the bone/bioadhesive graft delivery system serves as a sustained-release carrier for vancomycin and recombinant human bone morphogenetic protein-2, demonstrating good antibacterial performance and enhancing the osteoinductive activity and osteogenic microenvironment of calf bone granules, thereby promoting the repair of bone defects. Overall, this system offers a promising alternative for the fabrication of bone granules delivery system, demonstrating significant potential as a treatment option for open bone defects.

## Highlights

• The injectable delivery system uses bioadhesive to deliver the bone granules.
• The self-healing bone/bioadhesive delivery system allows bone particles to be evenly mixed within it.
• The bioadhesive has hemostatic ability, resulting from the reinforcement of bone granules.
• The bone/bioadhesice delivery system has the ability to deliver different functional additives.

## Graphical abstract

This scheme illustrates the application of PG/Van/B/CB bone/bioadhesive grafts delivery system in irregular bone defects. The release of Van from the system demonstrates antibacterial properties. Meanwhile, the release of rhBMP-2 and calf bone contributes to bone regeneration by enhancing the differentiation of BMSCs into osteoblasts, while the hydrogel's antioxidant properties protect BMSCs from oxidative stress. Hemostasis is achieved by injecting the system into hemorrhage sites, through its sealing properties.

**Figure:**
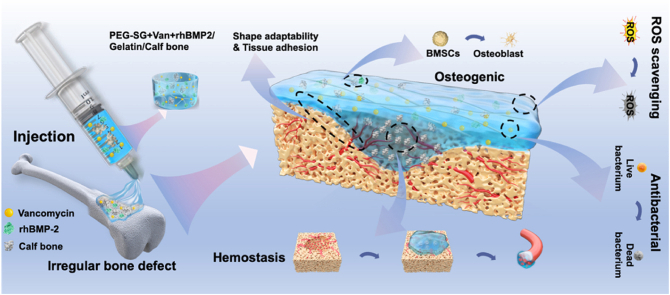

## 1 Introduction

With the rapid development of urban construction and transportation, the incidence of open bone defects resulting from high kinetic and potential energy injuries has significantly increased [1,2]. Open bone defects represent complex injuries that not only cause significant bone loss but also result in severe bleeding [3]. Bone grafting is widely recognized as the most effective treatment for the repair of critical bone defects [4], ranking as the second most commonly transplanted tissue after blood, leading to a substantial demand for both traditional and alternative bone grafting solutions in numerous countries. In the United States, over 500,000 patients undergo bone grafting procedures annually for various conditions, including fractures, bone tumors, joint fusions, and spinal fusions, with the estimated cost exceeding $3 billion [5].

Autologous bone grafting is widely regarded as the most effective treatment for critical-sized bone defects [6]. Considering the limited availability of autologous bone [2,7,8], other bone grafting materials are also widely used to substitute for autologous bone, including allografts, xenografts, and synthetic grafts [9,10]. However, neither autologous bone nor other bone substitute materials can be considered perfect bone graft options under their present formulations. These pre-formed bone grafts are typically processed into granules for improved application. However, the mismatched shapes between the bone granules and the bone defects site can easily create dead spaces, leading to delayed or non-healing of the bone defects [11,12]. Meanwhile, these bone granules can easily become dislodged from the filling site due to muscle pulling, flushing or blood flow [13]. Therefore, if the bone graft system could be adhered to the bone defects site, it would significantly improve the situation of displacement. Additionally, bone defects is frequently associated with substantial bleeding, which can be challenging to manage during treatment and lead to massive intraoperative and postoperative blood loss [[14], [15], [16]]. As a result, postoperative drainage of the blood is routinely employed, which significantly increased the complications such as hypotensive anesthesia, postoperative bedtime and patient's suffering [17]. Therefore, there is need to develop bond graft formulations that can fit and adhere to irregular shape of the defects area with the ability to promote hemostasis, which will significantly increase the therapeutic efficacy of traditional bone grafting.

Efforts are being made to address these challenges by integrating bone grafts with viscous carriers like collagen, chitosan, hyaluronic acid, and glycerol to produce diverse forms such as sponges, strips, injectable putty, and paste [2]. However, none of the existing materials can comprehensively tackle the issues mentioned, such as unstable retention post-application and inadequate hemostatic properties. Consequently, the development of novel carrier materials for various bone grafting substances is still needed.

Injectable bioadhesive are promising candidates for bone graft delivery. They can provide stable retention because of their inherent tissue adhesion, solving the problem of mismatched shapes and dislocation. They can adapt to every corner of the wounds resulting from its injectability [18,19]. However, the majority of traditionally injectable bioadhesive are of the AB type, which complicates the creation of a homogeneous mixture with bone grafts that tend to aggregate in solutions [20]. This leads to the failure to form uniform bone graft formulations for injection delivery. Since the uniformity of bone materials directly influences mechanical property and the state of bone regeneration, leading to bone delayed healing or bone nonunion [21]. One solution is to mix the AB components first and then blend the bone particles within the hydrogel through physical stirring. At this point, the system, due to an increase in viscosity, allows the bone particles to remain uniformly dispersed within the bioadhesive. However, this post-gelation mixing method is prone to damaging the network structure of the bioadhesive due to mechanical stirring, leading to a decline in bioadhesive's mechanical strength. Providing the bioadhesive with self-healing properties can address this issue. During the mixing process, the self-healing capability allows the network, disrupted by physical stirring, to repair itself, thereby maintaining the integrity of the bioadhesive's network structure. Hence, in this study, an injectable and self-healing bone grafts delivery system with strong sealing ability were fabricated with injectable and self-healing bioadhesive, prepared by Bi-armed Polyethylene Glycol Succinimidyl Glutarate (Bi-PEG-SG) and gelatin (PG hydrogel).

**Scheme 1:**
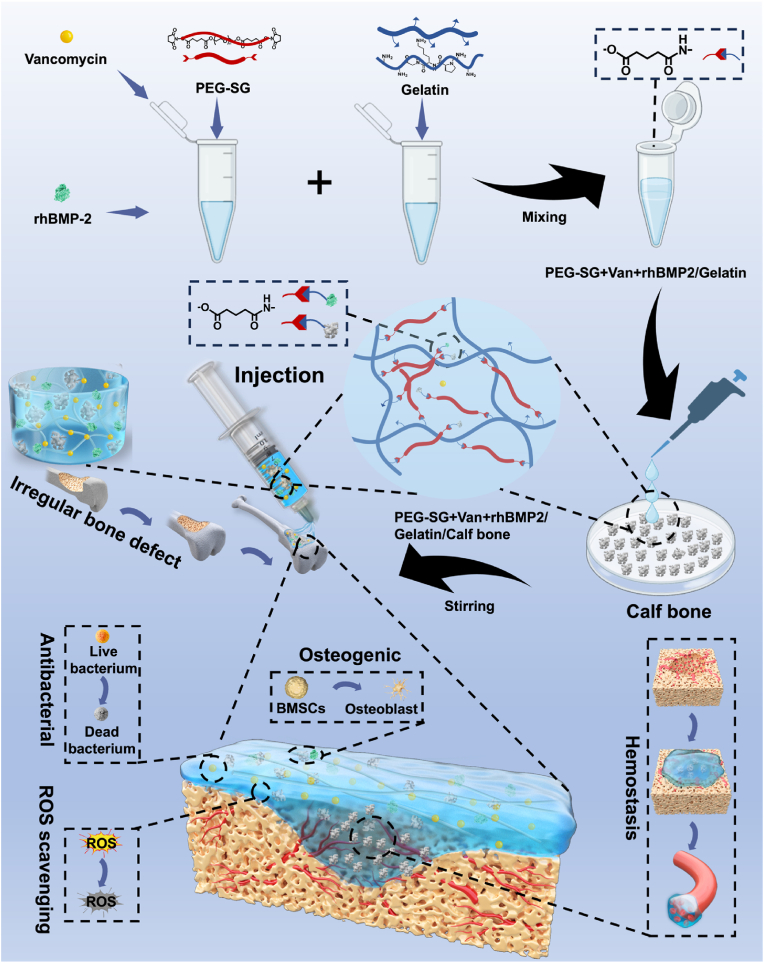
This scheme illustrates the preparation of the PG/Van/B/CB hydrogel system and its application in irregular bone defects. The PG/Van/B/CB hydrogel system is prepared by mixing Van and rhBMP-2 into a Bi-PEG-SG solution, which is then combined with gelatin. Following this, the mixture of Bi-PEG-SG/Van/B and gelatin is added to calf bone and subjected to further mixing and stirring before gelation. The release of Van from the system demonstrates antibacterial properties. Meanwhile, the release of rhBMP-2 and calf bone contributes to bone regeneration by enhancing the differentiation of BMSCs into osteoblasts, while the hydrogel's antioxidant properties protect BMSCs from oxidative stress. Hemostasis is achieved by injecting the system into hemorrhage sites, through its sealing properties.

Lyophilized calf bone(CB), a biocompatible and readily available graft material with excellent osteoconductivity [22,23], was chosen as the representative of the bone grafts(Scheme 1). With self-healing properties, the CB can be mixed after the gelation of the bioadhesive, allowing for a uniform mixture. Here, the tissue adhesion is achieved by the reaction between active ester carried by Bi-PEG-SG and tissue proteins. Meanwhile, type I collagen is one of the main components of CB [24]. Consequently, this system is reinforced through microstructural improvements and amidation reactions between CB and Bi-PEG-SG. These interactions serve to increase the cohesion strength of the bioadhesive, thereby enhancing its hemostatic efficacy [25]. Because of the gelatin used, the system exhibits antioxidant properties, which may be advantageous for bone regeneration [26]. Another function of the bioadhesive delivery system is that they can easily load different functional additives for bone grafts delivery. Open bone defects are complex injuries and carry a potential risk of infection due to the wound being exposed to the external environment [27,28]. Vancomycin (Van), commonly used as a prophylactic antibiotic for postoperative orthopedic infections, was loaded into the bone graft system. To further enhance osteoinductive properties of CB, recombinant human bone morphogenetic protein-2 (rhBMP-2, B) was added into the system to make PG/Van/B/CB to promote bone regeneration.

The performances of the resulting system, including self-healing capabilities, injectability, tissue adhesion, shape adaptability, functional additives release, antioxidant properties and antibacterial experiments, were evaluated. Then its hemostatic performances were evaluated in both the Sprague-Dawley (SD) rat femoral artery transection hemorrhage model and the New Zealand rabbit femoral defect hemorrhage model. In the subcutaneous heterotopic osteogenesis model in SD rats, the co-delivery of rhBMP-2 and CB improved the osteogenic microenvironment and facilitated bone regeneration. Additionally, the adherent PG hydrogel significantly enhanced the retention of CB granules at the bone defect site in the skull defect model, thereby facilitating the repair and reconstruction of the defect. By integrating its hemostatic and antibacterial functions, the PG/Van/B/CB bone/bioadhesive grafts delivery system, based on the bidirectional enhancement method, can be regarded as a bioactive material and its effects can change over time. It ensures complete bone hemostasis during the procedure, prevents infection of open bone defects in the early postoperative period, improves the osteogenic microenvironment in the long term, and promotes bone defects repair. In summary, the PG/Van/B/CB bone/bioadhesive grafts delivery system offers a novel solution for the complex clinical scenarios associated with open bone defects, which will also inspire the development of other bioadhesive system for bone grafts delivery.

## 2 Materials and methods

### 2.1 The preparation and characterization of bone/bioadhesive grafts delivery system

#### 2.1.1 Synthesis of Bi-PEG-SG

The synthesis of Bi-PEG-SG was performed according to our previous method. In summary, Bi-armed polyethylene glycol with a molecular weight of 6000 Da (Bidepharm, China), 4-dimethylaminopyridine (DMAP, Bidepharm, China), and glutaric anhydride (SA, Yuanye, China) were dissolved in methylene chloride and reacted at room temperature overnight. A 5% (w/w) NaCl solution was then added to the reaction mixture to remove impurities through shock and centrifugation, a process that was repeated four times. Anhydrous magnesium sulfate was employed to eliminate any residual water. Following the initial rotary evaporation, the reaction mixture was precipitated using petroleum ether and subsequently subjected to secondary rotary evaporation. The intermediate product, Bi-PEG-GA, was extracted, dried in a vacuum drying oven overnight, and then reacted with 1-ethyl-3-(dimethylaminopropyl) carbodiimide hydrochloride (EDC, Bidepharm, China) and N-hydroxysuccinimide in methylene chloride (NHS) at room temperature overnight. The purification steps were repeated, and the final product, Bi-PEG-SG, was obtained after lyophilization for three days and was stored as a dry powder in a freezer at 4 °C. Before use, it is important to allow the powder to reach room temperature before opening the storage tube. For application, the powder should be dissolved in PBS (0.2 M, pH = 4) to create the precursor solutions. Once the powder is fully dissolved in the liquid, it should be used within 30 min in conjunction with gelatin. The chemical structures were analyzed using ^1^H and ^13^C Nuclear Magnetic Resonance (NMR) spectroscopy (JEOL, Japan).

#### 2.1.2 Preparation of PG adhesive hydrogel

Bi-PEG-SG was dissolved in PBS (0.2 M, pH = 4) to achieve a final concentration of 10% (w/v), while gelatin (Type A, Sigma-Aldrich, Germany) was dissolved in PBS (0.2 M, pH = 9) at 55 °C to reach a final concentration of 20% (w/v). Here, PBS (0.2 M, pH = 9) was chosen to dissolve gelatin to expose more amino groups for crosslinking by reducing amino group protonation. PBS (0.2 M, pH = 4) was chosen to neutralize the pH of the final systems and prevent the deactivation of the active ester in Bi-PEG-SG. The PG adhesive hydrogels were formed by combining Bi-PEG-SG and gelatin in a 1:1 vol ratio. Van and rhBMP-2 were dissolved in PBS (0.2 M, pH = 4) containing 10% Bi-PEG-SG.

#### 2.1.3 Gelation test

The gelation time was determined using the vial tilting method. CB was weighed after being placed in a disc-shaped mold (diameter = 8 mm, height = 8 mm). Subsequently, PG hydrogel was injected into the mold. After gelation, the weight of the PG/CB was measured to analyze the weight ratio of the hydrogel system (CB:PG 9:20). The mixing of CB was divided into two steps. The first step involved preparing the PG/Van/B mixture. The second step consisted of adding the PG/Van/B mixture to pre-treated granules CB to create a homogeneous mixture through stirring. After the formation of these hydrogels, the samples were incubated at 37 °C for 15 min. No dissolution was observed, confirming the successful gelation of the samples. Gelation time was determined when the compounds no longer flowed upon tilting the vial. The hydrogel system was freeze-dried and observed under a scanning electron microscope (SEM, HITACHI, Japan).

#### 2.1.4 In vitro swelling ratio test

The PG and PG/CB hydrogels were injected into a 15 mL centrifuge tube, followed by the addition of 3 mL of PBS (0.01 M, pH 7.4) (n = 3). The tube was then placed on a rotational table set to a speed of 50 rpm and a temperature of 37 °C. The hydrogels were subsequently removed and gently dried with filter paper. They were weighed at specified time intervals (4 h, 8 h, 12 h, 24 h, 36 h, and 48 h). The swelling ratio (SR) was calculated using the formula: SR = (W2 - W1) × 100%, where W2 represents the weight at a specific time and W1 represents the initial weight.

#### 2.1.5 In vitro and vivo degradation test

Standard degradation experiment in *vitro*: The PG and PG/CB hydrogels were injected into a 15 mL centrifuge tube, followed by the addition of 3 mL of PBS (0.01 M, pH = 7.4) (n = 3). The tube was then placed on a rotational table set to a speed of 50 rpm and a temperature of 37 °C. Subsequently, the hydrogels were removed and gently dried using filter paper. The weights of the remaining hydrogels were measured at specific time intervals. Finally, the degradation products were analysis through advanced polymer chromatography at different time points.

Degradation experiment in an enzymatic environment in *vitro*: The PG and PG/CB hydrogels were injected into a 15 mL centrifuge tube, followed by the addition of 3 mL of PBS (0.01 M, pH = 7.4) with a gelatinase solution containing 2 U/mL.

Degradation experiment *in vivo*: SD rats weighing between 200 and 220 g were used in this study. A 3 mm femoral defect was created using a trephine drill. The surgical site was flushed with normal saline, and the PG hydrogel (labelling by ICG-NHS) was placed in the defect. The skin was then sutured with 3.0 non-absorbable sutures and the fluorescence was observed using an IVIS Spectrum *in vivo* imaging system to indicate the degradation (Vieworks, Korea).

#### 2.1.6 Self-healing ability and mechanical properties test

The PG and PG/CB adhesive hydrogels were prepared and injected into molds to create disc-shaped hydrogels (diameter = 10 mm, height = 6 mm). After 5 min, two discs were produced for each hydrogel, with one disc stained with methylene blue. Subsequently, the disc-shaped hydrogels were crushed, and halves of each crushed hydrogel were mixed in disc molds. The molds were sealed with parafilm and incubated at 37 °C for 1 h before the hydrogels were removed for observation.

#### 2.1.7 Compression property test

The PG and PG/CB hydrogels were initially prepared in cylindrical shapes with a diameter of 8 mm and a height of 8 mm. After 10 min, the hydrogel was removed, cut into two pieces, and then reconnected at the fracture site. The molds were sealed with parafilm and incubated at 37 °C for 1 h. Subsequently, the rejoined hydrogels within the molds were subjected to a compression test (20%, 40%, 50%) using a universal tension machine (CMT1103, SASCK, China) at a speed of 5 mm/min.

#### 2.1.8 Tensile property test

3 cm strip-shaped hydrogels were formed by injecting the PG and PG/CB adhesive hydrogels into molds. After a 10-min incubation period, two strips were produced from each hydrogel. One strip was dyed with methylene blue, while the other was dyed with rhodamine. These strips were then halved, and the halves from different strips were reconnected and incubated at 37 °C for 1 h. Subsequently, the repaired hydrogel in the mold underwent a stretching test using a universal tension machine (CMT1103, SASCK, China) at a speed of 5 mm/min.

#### 2.1.9 Injectable property and shape suitability

CB (Dasting, China), femur of rabbit and artificial bone (self-cured calcium phosphate, Rebone, China) was placed into a syringe, followed by the injection of 1 mL at room temperature. Subsequently, the PG/CB hydrogel was immersed in double-distilled water before gelation to observe its morphology. Moreover, the hydrogels can be injected into molds of various shapes using syringe.

To enhance the delivery controllability and clinical applicability of PG/CB delivery system adhesive, we conducted extra injection strength experiments using a homemade pneumatic injection system. As shown in Fig. S4, this system consists of three key components: a power module (vacuum pump, pressure gauge, air filter), a gelation module and a mixing valve), and a delivery module (adhesive delivery channel). The system enables precise control over injection parameters, including air flow rate (via a regulator) and ejection pressure (monitored in real-time by the pressure gauge). Subsequently, *in vitro* experiments were performed to evaluate the delivery efficacy of the PG/CB delivery system using the homemade pneumatic injection system.

#### 2.1.10 Adhesion strength test

Adhesion strength tests were carried out on porcine skin samples that were cut into 25 mm ∗ 10 mm rectangles. Each sample was treated with either 40 μL of PG or PG/CB hydrogels, overlapped (10 mm ∗ 10 mm), and pressed together under a 100 g weight for 30 min at 37 °C. Four replicate samples were prepared for each formulation. The adhesive strength was evaluated using an electronic universal testing machine (CMT1103, SASCK, China) at a speed of 5 mm/min.

Bone adhesion strength tests were carried out on porcine bone, the porcine bone was cut, and PG and PG/CB were injected into the surface. After incubation for 15 min, the adhesion tests were performed, using a universal tension machine (CMT1103, SASCK, China) at a speed of 5 mm/min.

#### 2.1.11 Van and rhBMP-2 release kinetics of PG/van/CB

The PG/Van/CB hydrogel system was placed in a 24-well plate containing 2 mL of PBS (0.01 M, pH = 7.4) per well and incubated at 37 °C with shaking at 50 rpm. Supernatant samples were collected at specific time intervals, and the concentrations of Van in these samples were quantified using HPLC.

This PG/B/CB hydrogel system was placed in a 24-well plate with 2 mL of PBS (0.01 M, pH = 7.4) in each well and incubated at 37 °C with shaking at 50 rpm. The supernatant was collected at specific time points, and the concentrations of rhBMP-2were measured using ELISA kits and calculating cumulative releases and fitting release curves.

### 2.2 Sealing *in vitro* and *in vivo* hemostatic of bone/bioadhesive grafts delivery system

#### 2.2.1 Bursting pressure test

3 cm ∗ 3 cm porcine skin sample was selected to evaluate the bursting pressure of a specific device. A 3 mm hole was precisely punched in the center of the skin, and two types of hydrogels, PG and PG/CB, were used as sealants. The testing area was confined to a model with a diameter of 10 mm and a height of 6 mm. After allowing the hydrogel to stabilize for 10 min, it was positioned on the measuring instrument. Pressure was gradually increased using a syringe pump at a rate of 5 mL/min until the hydrogel ruptured, and the maximum pressure value was recorded. In addition to PG and PG/CB, fibrin glue (BIOSEAL BIOTECH, Johnson & Johnson) was used, with its main components being fibrinogen and thrombin. Meanwhile, fibrin glue/CB was also included as control groups in the experiment. The assay was repeated six times for each sample.

#### 2.2.2 Femoral artery hemostasis test in SD rats

The SD rats was used to build a massive hemorrhage model. After the rats was anesthetized, the femoral artery was exposed and the proximal and distal ends of the femoral artery are clamped using hemoclips, and then clipped through the center. Then, the PG, PG/CB and Fibrin glue was injected in situ at femoral artery. Ten minutes later, the hemoclips were removed, observing bleeding and recording the amount and time.

#### 2.2.3 Femoral condyle defect hemostasis test in New Zealand rabbits

Briefly, the rabbits were anesthetized, and the distal femur was shaved and disinfected. After cutting the skin and subcutaneous tissue, a cylindrical defect 6 mm in diameter was created. Then the CB, PG/CB, Bone wax was injected in the bone defect, observing bleeding and recording 10 min of bleeding amount.

### 2.3 Antioxidant of bone/bioadhesive grafts delivery system

#### 2.3.1 ABTS + radical scavenging assay

The 2,2′-azinobis-(3-ethylbenzthiazoline-6-sulphonate) (ABTS+) radical solution was prepared by mixing equal volume 7.4 mM ABTS radical solution with 2.45 mM potassium persulfate solution, incubating in the dark for 12 h at 25 °C. 50 μL of ABTS radical stock solution was added to 3 mL deionized water for dilution. Different masses hydrogels of (0, 5, 10, 20, and 40 mg) and ascorbic Vitamin C (Vc) (0.5 mg/mL, 50 μL) were added to 3 mL of diluted ABTS radical solutions, which were then incubated for 10 min at 37 °C in the dark. The absorbance at OD 734 nm was measured using a multiscan spectrum (SuPerMax 3100, Shanpu, China). ABTS+ radical scavenging activity was calculated according to the following equation

ABTS + radical scavenging ratio (%) = [(A0 - A1)/A0] × 100

where A0 is the absorbance of the control (0 mg), and A1 is the absorbance of the sample and Vc groups.

#### 2.3.2 DPPH radical scavenging assay

DPPH (2,2-diphenyl-1-trinitrohydrazyl) radical solution was prepared at a concentration of 0.1 mg/mL by dissolving DPPH in methanol. Subsequently, varying amounts of samples were added to 3 mL of the DPPH radical solution: PG (40, 70, and 100 mg), PG/Van (100 mg), PG/Van/B (100 mg; Van at 0.5 mg/mL and B at 1 μg/mL), and Vc at a concentration of 0.5 mg/mL (50 μL), with Vc serving as the positive control. The mixture was incubated in the dark at room temperature for 20 h. The absorbance of the mixture at 517 nm was then measured using a multiscan spectrophotometer. The DPPH radical scavenging activity was calculated using the formula: DPPH radical scavenging ratio (%) = [(A0 - A1)/A0] × 100, where A0 represents the absorbance of the control (0 mg) and A1 represents the absorbance of the samples and Vc groups. Each experiment was conducted in triplicate.

#### 2.3.3 Hydroxyl radical scavenging assay

·OH (Hydroxyl radicals) were generated in a Fenton-type reaction. A stock solution of hydroxyl radicals was prepared by mixing equal volumes of 9 mM salicylic acid ethanol solution, an aqueous solution of 9 mM ferrous sulfate, and an aqueous solution of 8.8 mM hydrogen peroxide. Dilution was carried out by adding 1.5 mL of distilled water to 1.5 mL of the stock solution. PG (10, 30, and 50 mg), PG/Van 50 mg, PG/Van/B 50 mg (Van 0.5 mg/mL, B 1 μg/mL), and Vc (Vc 2 mg/mL, 160 μL) were then added to 3 mL of the diluted hydroxyl radical solution, with Vc serving as the positive control. The mixture was incubated at 25 °C for 10 min in the dark before centrifugation (5000 r/min, 4 min). Subsequently, the absorbance at 510 nm was measured using a multiscan spectrum. The hydroxyl radical scavenging activity was determined using the equation: Hydroxyl radical scavenging ratio (%) = [(A0 - A1)/A0] × 100, where A0 represents the absorbance of the control (0 mg) and A1 represents the absorbance of the sample and Vc group. Each experiment was conducted in triplicate.

Finally, 3 mL of ABTS+, DPPH, and ·OH were taken separately and incubated in the dark with 200 mg of PG hydrogel for 10 min (ABTS+), 20 h (DPPH) and 10 min (·OH). After incubation, 30 μL of the sample was taken and mixed with 30 μL of 100 mM 5,5-Dimethyl-1-Pyrroline-N-Oxide (DMPO), using deionized water as the solvent. After thorough mixing, the mixture was drawn using a capillary tube, placed in a quartz tube, and then inserted into the EPR (Electron Paramagnetic Resonance) resonator cavity for testing the capture of free radicals in the aqueous system by DMPO, including ABTS+, DPPH, and ·OH.

#### 2.3.4 Scavenging of intracellular ROS free radical

The study evaluated the scavenging of intracellular Reactive Oxygen Species (ROS) free radical ability of PG with NIH 3T3 mouse fibroblasts. Initially, the fibroblasts were seeded in 48-well plates at a density of 3 × 10^4^ cells per well and incubated at 37 °C with 5% CO_2_ overnight to allow for adhesion. In the experiment, we selected a reactive oxygen species (ROS) positive control drug (Rosup) to induce oxidative stress in NIH3T3 cells. The levels of intracellular ROS under oxidative stress conditions were directly assessed using the 2,7-dichlorodihydrofluorescein diacetate (H2DCFDA, Beyotime, China) as fluorescent probe, where green fluorescence indicates the presence of ROS.

#### 2.3.5 Antioxidant experiments with NIH 3T3 cells

The study evaluated the protective effects of PG against oxidative stress in NIH 3T3 mouse fibroblasts. Initially, the fibroblasts were seeded in 48-well plates at a density of 3 × 10^4^ cells per well and incubated at 37 °C with 5% CO_2_ overnight to allow for adhesion. Subsequently, tert-butyl hydroperoxide (tBOOH, 100 μM) was added to induce oxidative stress by mixing it with the cell culture medium for 20 min. Different doses of PG (20, 30, and 50 mg), PG/Van (50 mg), and PG/Van/B (50 mg; Van at 0.5 mg/mL, B at 1 μg/mL) were then introduced to the mixture. After 5 h in the cell incubator, the metabolic activity was assessed using the Cell Counting Kit-8 (CCK-8, Biosharp, China) and a live/dead cell staining kit (Solarbio, China).

### 2.4 Biocompatibility of bone/bioadhesive grafts delivery system

#### 2.4.1 Cytocompatibility test

The cytocompatibility of PG/G/Van/B was evaluated using NIH 3T3 mouse fibroblasts with the Cell Counting Kit-8 (CCK-8) and live/dead cell staining. Prior to testing, the raw materials and PG/Van/B hydrogel underwent sterilization under UV light overnight. Subsequently, 200 mg of PG/Van/B was incubated in 10 mL of cell culture medium for 12 h to produce the leachate (1× at 20 mg/mL), which was then further diluted to concentrations of 10×, 100×, and 1000×. Another 200 mg of PG/Van/B was degraded in 10 mL of cell culture medium to obtain the degradation product solution (20 mg/mL), which was then diluted to various concentrations (2 mg/mL, 0.2 mg/mL, and 0.02 mg/mL). NIH 3T3 mouse fibroblasts were seeded in 96-well plates at a density of 3 × 10^4^ cells per well and incubated at 37 °C with 5% CO_2_ overnight. After cell adhesion, the culture medium was replaced with varying concentrations of the leachate and degradation product solutions, or the cells were co-cultured with solid PG/Van/B hydrogel (50 mg, 20 mg, 10 mg, and 5 mg). Following incubation for 24 and 48 h, 10% CCK-8 solution was added to each well and incubated at 37 °C with 5% CO_2_ for 2 h. The absorbance of the samples was measured at 450 nm using a multiscan spectrophotometer (SuPerMax 3100, Shanpu, China). Metabolic activity was calculated using the formula: Metabolic activity (%) = [(As - Ab)/(Ac - Ab)] × 100, where As represents the absorbance of the experimental group, Ac is the absorbance of the control group, and Ab is the absorbance of the blank group. Each experiment was repeated three times. Metabolic activity above 70% was considered non-cytotoxic. Cytotoxicity was evaluated using live/dead cell staining. Following 24 and 48 h of incubation, the leaching content, degradation solution, and PG/Van/B was removed, and the cells were washed twice with PBS (0.01M, pH = 7.4). Live/dead cell staining was then performed according to the provided instructions and observed using an inverted fluorescence microscope.

#### 2.4.2 In vitro hemolysis test

Fresh blood from SD rats (2 mL) was diluted with normal saline (20 mL) and centrifuged at 1200 rpm for 15 min. The supernatant was discarded after each round of centrifugation. The resulting erythrocyte suspension was then diluted to a 2% (V/V) concentration with normal saline. The experimental group (W1) included leaching content CB, PG/CB, PG/CB/Van, and PG/Van/B/CB (0.5 mL each) mixed with the erythrocyte suspension (0.5 mL). The positive control (W0) consisted of deionized water (0.5 mL) mixed with the erythrocyte suspension (0.5 mL), while the negative control (W2) involved normal saline (0.5 mL) mixed with the erythrocyte suspension (0.5 mL). All samples were incubated at 37 °C for 1.5 h and centrifuge for 5min. The supernatant from the centrifuged samples was then transferred to a 96-well plate for absorbance measurement at a detection wavelength of 540 nm. The hemolysis rate was calculated using the formula: hemolysis ratio (%) = (W1 - W2)/(W0 - W2) × 100.

#### 2.4.3 In vivo biocompatibility test

SD rats weighing between 200 and 220 g were used for the study. Subcutaneous pockets were created by making a 1 cm incision on the dorsal skin (5 cm from the connecting line of both ears and 1 cm from the midline of the dorsum). The CB, PG/Van/CB, and PG/Van/B/CB were placed in the left dorsum of the rats (n = 4). The incisions were closed with 3.0 non-absorbable sutures, and the rats were returned to their cages for recovery. After 4 weeks, the rats were euthanized by cervical dislocation. Critical organs such as the heart, liver, spleen, lungs, and kidneys were harvested for H&E staining.

### 2.5 Bacteriostasis of bone/bioadhesive grafts delivery system

Van was dissolved in a PBS (0.2 M, pH = 4) containing 10% Bi-PEG-SG, with concentrations of 2 mg/mL, 1 mg/mL, 0.4 mg/mL, and 0.2 mg/mL. Additionally, gelatin was dissolved in a PBS (0.2 M, pH = 9) and heated to 55 °C to achieve a final concentration of 20% (w/v). The antibacterial hydrogels were prepared by mixing Van/Bi-PEG-SG with gelatin at a volume ratio of 1:1 to obtain concentrations of 1 mg/mL, 0.5 mg/mL, 0.2 mg/mL, and 0.1 mg/mL.

#### 2.5.1 In vitro bacteriostasis ratio test

The concentration of *S.aureus* was adjusted to 10^5^ CFU/mL in a PBS (0.01 M, pH = 7.4) solution. Different concentrations of PG/Van antibacterial hydrogels were added to a 24-well plate, with each well containing 1 mL of bacterial suspension, and then incubated at 37 °C for 24 h. After incubation, the bacterial suspension was diluted by 100-fold, and the viable bacteria were counted using the plate counting method (n = 3). The PG/Van/CB bacteriostasis ratio test was conducted in the same manner as described above.

#### 2.5.2 In vitro inhibition zone assay

In this assay, *S.aureus* was employed as the test microorganism. The bacterial solutions were inoculated into Tryptic Soy Broth (TSB) and incubated at 37 °C for 24 h, reaching a concentration of 10^7^ Colony Forming Units per milliliter (CFU/mL). Subsequently, 100 μL of the bacterial solution, containing 10^6^ CFU, was evenly spread onto TSB agar plates. Cylindrical hydrogels composed of PG/Van (diameter = 10 mm, height = 5 mm), which had been pre-irradiated with ultraviolet light overnight, were placed at the center of the agar plates containing the bacteria. Following a 24-h incubation at 37 °C, the diameter of the inhibition zone was measured using a steel ruler. Each experiment was conducted in triplicate. The PG/Van/CB bacteriostasis ratio test was conducted in the same manner as described above.

#### 2.5.3 In vivo antibacterial test

SD rats weighing between 200 and 220 g were used in the experiment. The rats’ back hair was shaved and sterilized with iodophor. Subcutaneous pockets were created by making a 1 cm incision on the dorsal skin, located 5 cm from the line connecting both ears and 1 cm from the midline of the dorsum. Cylindrical PG/Van hydrogels (diameter = 10 mm, height = 6 mm), which had been pre-irradiated with ultraviolet light overnight, were implanted in the left dorsum of the rats, with a bacterial suspension injected at the implantation site (n = 3). A control group received bacterial suspensions injected subcutaneously. After a 7-day period, the rats were euthanized via cervical dislocation. The soft tissue surrounding the hydrogel was collected, fixed with 4 % paraformaldehyde, and examined histologically using H&E staining.

### 2.6 Angiogenic and osteogenic capacity *in vitro* of bone/bioadhesive grafts delivery system

#### 2.6.1 Scratch assay of HUVECs

HUVECs were cultured in 96-well plates at a density of 3 × 10^4^ cells/mL. Once the cells reached 80–90% confluence, a scratch was made using a 200-μL pipette tip. The cells were then cultured with the leachates of PG, PG/CB, and PG/B/CB in a serum-free medium. Images of migrated HUVECs were randomly captured at 0 h and 24 h. ImageJ software was used to calculate the migration area. The migration ratio of HUVECs was determined using the formula: migration rate (%) = [(A0 - An)/A0] × 100%, where A0 and An represent the initial scratch area and the remaining area after migration, respectively.

#### 2.6.2 Transwell assay of HUVECs

HUVECs were seeded at a density of 3.0 × 10^4^ cells/mL in serum-free medium in the upper chamber of 24-well transwell plates with 8-μm pore filters. Each well contained one specimen and was incubated for 24 h in serum-free medium. The leachates of PG, CB, and PG/B/CB in medium containing 10% Fetal Bovine Serum (FBS) were added to the bottom chamber. After incubation, cells attached to the upper surface of the filter membranes were removed using a wet cotton swab, and the invaded cells were fixed with 4% paraformaldehyde (PFA) for 10 min. The cells were then stained with 0.1% crystal violet solution for 60 min and imaged under an inverted microscope. Cell numbers were quantified using ImageJ software.

#### 2.6.3 Tube formation assay HUVECs

A 96-well culture plate was coated with 50 μL of Matrigel and incubated at 37 °C for 30 min to facilitate gelation. HUVECs were then seeded onto the Matrigel-coated (Corning, USA) plate at a density of 2 × 10^4^ cells per well. After 6 h of incubation, the tubule network formed by HUVECs was visualized using an inverted microscope. The resulting images were analyzed with ImageJ to quantify the number of branches and the total length of the tubes generated by the HUVECs.

#### 2.6.4 Transwell assay of MC3T3-E1

MC3T3-E1 were seeded at a density of 3.0 × 10^4^ cells/mL in serum-free medium in the upper chamber of 24-well transwell plates with 8-μm pore filters. Each well contained one specimen and was incubated for 24 h in serum-free medium. The leachates of PG, CB, and PG/B/CB in medium containing 10% Fetal Bovine Serum (FBS) were added to the bottom chamber. After incubation, cells attached to the upper surface of the filter membranes were removed using a wet cotton swab, and the invaded cells were fixed with 4% paraformaldehyde (PFA) for 10 min. The cells were then stained with 0.1% crystal violet solution for 60 min and imaged under an inverted microscope. Cell numbers were quantified using ImageJ software.

#### 2.6.5 Alkaline phosphatase colorimetric and activity assay

MC3T3-E1 cells were seeded in 24-well plates at a density of 2 × 10^4^ cells per well. The culture medium was supplemented with 5% penicillin-streptomycin and 10% FBS in α-MEM to establish standard cell culture conditions. After three days of culture, when the cells reached 70-80% confluence, they were divided into PG, PG/CB PG/B/CB groups.

MC3T3-E1 cells were cultured in differentiation media for 14 days, with media changes every 2 days. This process involved washing the cells with PBS (0.01 M, pH = 7.4), fixing them in 95% alcohol for 15 min at room temperature, incubating them with BCIP/NBT for 30 min, and then removing any unincorporated BCIP/NBT with distilled water. Microscopic images were captured using an inverted microscope.

After three washes with PBS (0.01 M, pH = 7.4), 200 μL of cell lysis buffer was added to each well to lyse the cells for 40 min. Subsequently, all samples were tested using an ALP kit. The samples were incubated with the reagent in a 37 °C water bath for 15 min, followed by the addition of a chromogenic agent. The optical density (OD) of each well was measured at a wavelength of 520 nm. The protein concentration of each sample was determined using the BCA kit (Sangon Biotech, China). ALP activity in each sample was calculated using the following formula: ALP activity = (MOD - BOD/SOD - BOD) ∗ (PSC - MSPC), where MOD and BOD represent the measured OD value and the blank OD value, SOD represents the standard OD value, and PSC and MSPC represent the phenolphthalein standard concentration and the measured sample protein concentration, respectively.

#### 2.6.6 qRT-PCR analysis

RNA was extracted from the cells of each group after 14 days using Trizol. The extracted RNA was then reverse transcribed into cDNA with the PrimeScript™ RT Reagent Kit, followed by amplification using the ABI 7500 fluorescent PCR system. The expression levels of osteogenic-related genes were determined using the 2−ΔΔCt method, where Ct values were normalized to β-actin within the same sample and further normalized to the Ct values of the control group on day seven. The primer sequences of the target genes are provided in Table S2.

#### 2.6.7 Immunofluorescence of OPN, OCN, RUNX2, Col1

After 14 days of culture in differentiation media, MC3T3-E1 cells were fixed with 4% paraformaldehyde, permeabilized with 0.5% Triton X-100, and blocked with 1% BSA. Subsequently, the cells were washed three times with PBS (0.01M, pH = 7.4), stained overnight at 4 °C with primary antibodies against OPN, OCN, RUNX2, and Col1, and then incubated with a secondary antibody for 2 h at room temperature. Following this, DAPI staining solution was added, and the cells were incubated for an additional 15 min before being photographed under a fluorescence microscope.

### 2.7 Osteogenic and angiogenic capacity *in vivo* of bone/bioadhesive grafts delivery system

#### 2.7.1 Ectopic bone-forming model

SD rats weighing between 200 and 220 g were utilized in the experiment. Subcutaneous pockets were formed by creating a 1 cm incision on the dorsal skin (5 cm from the connecting line of both ears and 1 cm from the midline of the dorsum). The CB, PG/Van/CB, and PG/Van/CB/B were implanted in the left dorsum of the rats (n = 3). The incisions were sutured with 3.0 non-absorbable sutures, and the rats were then returned to their cages for recovery. After 4 weeks and 8 weeks post-implantation, the rats were euthanized using cervical dislocation. Subcutaneous tissues were harvested for analysis using a micro-computed tomography machine. Subsequently, immunofluorescence staining for CD31 and HIF1 was conducted, along with H&E and Masson stainings.

#### 2.7.2 Evaluation of PG/van/B/CB for skull defect repair in rats

SD rats weighing between 200 and 220 g were used in this study. The rats were randomly assigned to four groups (n = 4). A midline incision was made on the cranium, exposing the skull bone after reflecting the skin and periosteum. A 5 mm transosseous critical-sized defect was created on the cranium using a trephine drill. The surgical site was flushed with normal saline, and the CB, PG/Van/CB, PG/Van/B/CB was placed in the defect. The periosteum and skin were then sutured with 3.0 non-absorbable sutures. The rats were allowed to heal for 4 and 8 weeks, the rats were euthanized using cervical dislocation, and the skull bone were collected for analysis using a micro-computed tomography machine, as well as H&E and Masson trichrome staining.

#### 2.7.3 Micro-CT analysis

After fixation with 4% paraformaldehyde, the sample was scanned using a high-resolution micro-CT system (Bruker, Skyscan). Subsequently, images were acquired for 3D reconstruction with the 3D Creator software. Parameters associated with new bone formation, such as bone mineral density (BMD), bone volume (BV/TV, BS/BV), and trabecular characteristics (Tb.N, Tb.Sp, Tb.Th), were calculated and analyzed using Mimics software

#### 2.7.4 Histological analysis

Histological Evaluation: Pathological sections of all experimental samples were evaluated using H&E and Masson trichrome staining. The skull and femur bones were fixed with 4% paraformaldehyde, decalcified with 5% EDTA, embedded in paraffin, sectioned at a thickness of 5 μm.

For H&E staining procedure: firstly, hematoxylin staining is performed to enhanced nuclear staining affinity, followed by treatment with an acidic ethanol differentiation solution (1% HCl - 70% ethanol) for 30 s, and then neutralized with a basic blueing solution (0.2% ammonia) to enhance the staining contrast. Then, eosin counterstaining is carried out, followed by dehydration and mounting for preservation. For Masson staining, firstly, the paraffin sections are immersed in Masson A solution to ensure that the nuclear chromatin sufficiently binds with the metal ions. Then, they are immersed in the mixed solution of Masson B and C, which enhances the differential staining of collagen and muscle fibers. Finally, the sections are immersed in Masson D solution, where hydrogen bonds specifically bind to the carboxyl groups of collagen fibers, resulting in a characteristic blue signal. The sections are then decolorized in a 1% acetic acid aqueous solution, followed by graded dehydration in an ethanol-xylene solution before mounting for preservation and examination under an optical microscope.

#### 2.7.5 Statistical analysis

The results between two groups were analyzed using a paired T-test, while the results among three groups were analyzed using a one-way analysis of variance (ANOVA) with Tukey-Kramer multiple comparison analysis. The statistical analysis was performed using SPSS 26.0. Data are presented as mean ± SD, and each experiment was repeated at least three times. The p-value of less than 0.05 was considered statistically significant (∗*P* < 0.05, ∗∗*P* < 0.01, ∗∗∗*P* < 0.001).

## 3 Results and discussion

### 3.1 Preparation and characterization of PG hydrogels and PG/van/B/CB

**Fig. 1:**
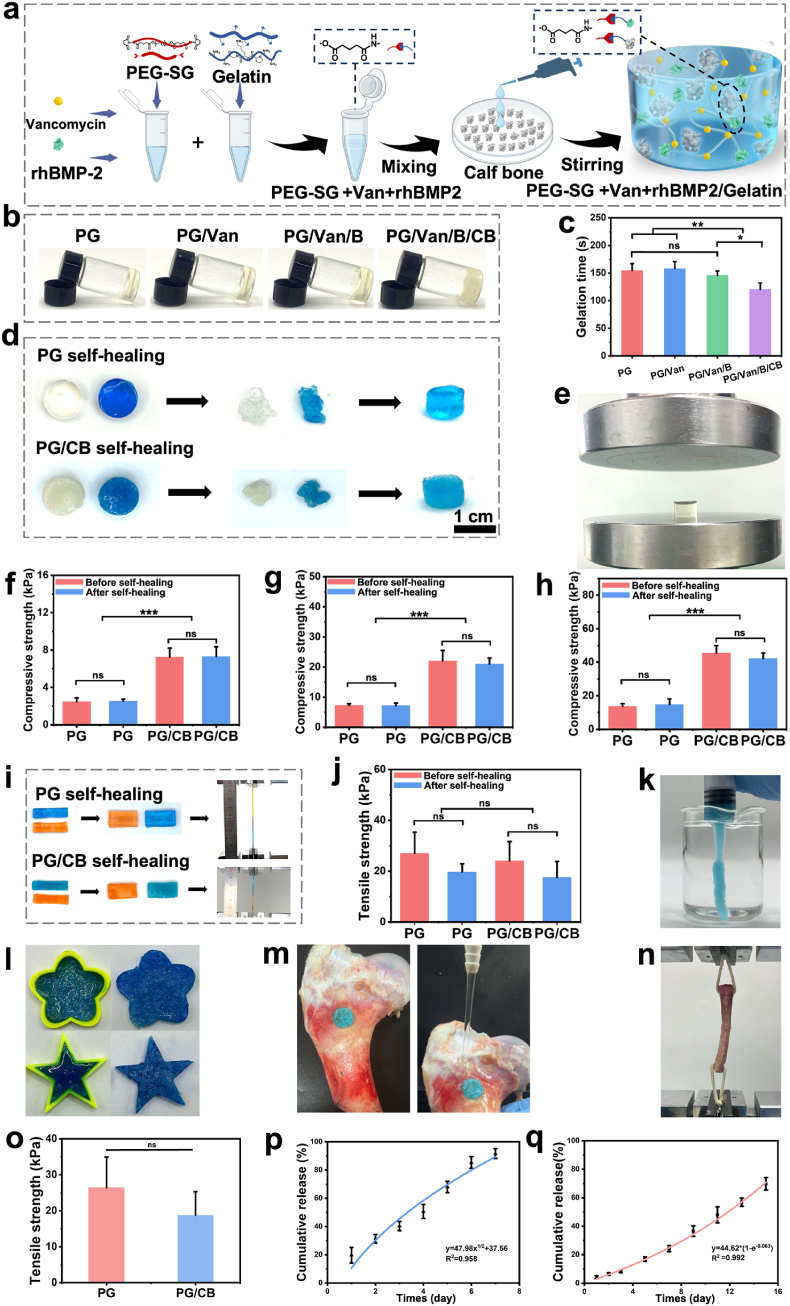
The bone/bioadhesive system showed properties. a) Schematic showing the preparation of PG/Van/B/CB hydrogel system. b) Preparation of PG, PG/Van, PG/Van/B hydrogel and PG/Van/B/CB system with the same volume and measure gelation time. c) Gelation time of PG, PG/Van, PG/Van/B and PG/Van/B/CB system. d) The performance of self-healing properties of PG and PG/CB delivery system when made into disc (Scale bar = 1 cm). e) Test compression property of before self-healing and after self-healing test using a universal tension machine at a speed of 5 mm/min (f, g, h) After self-healing at strain of 20% (f), 40% (g), and 50% (h) compared with those before self-healing (n = 3, mean ± SD, ns: not significant). i) The performance of self-healing properties of PG and PG/CB delivery system when made into strip and using a universal tension machine at a speed of 5 mm/min to test the tensile strength. j) Tensile strength of PG and PG/CB delivery system before and after self-healing. (n = 3, mean ± SD, ns: not significant). k) Presentation of the injectability of PG/CB delivery system. l) Presentation the shape adaptability PG/CB delivery system. m) Stable adhesion even under water flow washing. n) Lap shear tests were performed with bone tissue. o) The results of lap shear tests. p, q) The release of Van (p) and rhBMP-2 (q) of PG/CB delivery system, along with their corresponding fitted release kinetics curves.

Here, the bioadhesive (PG hydrogels) were chosen as the carriers for bone granules, which were inspired by our previous research [29,30]. PG hydrogels were synthesized by combining Bi-PEG-SG (Fig. S1 and S2). Another function of the bioadhesive is that they can easily load different functional additives. Here, to endow the system with antibacterial and bone regeneration promotion properties, Van and rhBMP-2 were first dissolved together with Bi-PEG-SG, and then the adhesive hydrogel was formed by mixing these solutions with gelatin (Fig. 1a). The final pH of the gel was measured to be 6.2 ± 0.1 using a flat pH meter [29]. The SEM images demonstrated that PG hydrogels were porous with the average pore size of 199.76 ± 80.0 μm (Fig. S3a) ranging from ∼100 μm to ∼400 μm (Fig. S3b). The porous structure will be allow osteoprogenitor cells (such as mesenchymal stem cells), osteoblasts, endothelial cells, and their protrusions to migrate into the scaffold and achieve *in vivo* host cell integration, new bone formation, and vascularization [[31], [32], [33]].

The gelation time was initially evaluated (Fig. 1b) and indicated that the addition of both Van and rhBMP-2 did not significantly affect the gelation time. (PG: 154.7 ± 12.22 s; PG/Van: 158.0 ± 12.77 s; PG/Van/B: 146 ± 7.94 s, Fig. 1c), despite both containing -NH2 groups. This phenomenon can be attributed to the insignificant representation of Van and rhBMP-2 in the PG/CB/B system, as they contain fewer amino themselves. It is worth noting that grafting BMP-2 to scaffolds through its amino groups have been widely used to increase its retention and circulation half-life, without influencing BMP-2's activity [[34], [35], [36]]. As a result, it is believed that here the PEGylation of BMP-2 would not influence its bioactivity. In the future, Van and rhBMP-2 can be changed to other functional additives for tailored applications. Meanwhile, when employing other compounds containing amine groups, it is essential to first assess whether the consumption of these groups could affect their biological activity.

In this study, lyophilized calf bone (CB, purchased from Dasting (Tianjin, China)) was chosen as a representative of traditional bone grafts. CB granules is commonly used as a bone graft material in clinical practice, which retains most of its organic components after defatting, decalcification, and freeze-drying, with type I collagen accounting for 90% of the organic matrix [24]. The SEM image (Fig. S3c) showed the average of the CB granule size used in this work was 19.02 ± 7.95 μm. Generally, for traditional injectable hydrogels, CB should be mixed with one of the precursor components. However, considering that CB, in the granules form, tends to settle easily in solution, leading to the uneven distribution of the bone granules. Therefore, the mixing of CB was divided into two steps. The first step is to prepare the PG/Van/B mixture to increase the viscosity of the system. Then, the second step involves adding viscous PG/Van/B to pre-treated granules CB to create a homogeneous mixture through stirring (Fig. 1a and b). This is due to that the increased viscosity prevents the aggregation of CB granules. Finally, the resulting mixture subsequently gelled completely. A significant reduction in gelation time was observed in the PG/Van/B/CB system (120.3 ± 12.01 s, *P* < 0.05, Fig. 1c). This reduction can be attributed to the increased viscosity after addition of CB granules, which limits the fluidity in the system. Additionally, the -NH_2_ groups present in type I collagen can react with the activated -COOH groups of Bi-PEG-SG in the hydrogel, leading to accelerated gelation.

To demonstrate the uniform distribution of the CB particles in the PG matrix, SEM and Energy Dispersive Spectrometer (EDS) were performed following previous report [37,38]. Firstly, the SEM demonstrated that CB particles (yellow) are evenly distributed on the surface and cross-section of PG hydrogel (Fig. S3d and S3e). Furthermore, the calcium even distribution (characteristic element of the CB particles in the bone/bioadhesive grafts delivery system, Fig. S3f and S3g) indicated that the CB particles are uniformly distributed within the hydrogel system.

Following the preparation of the system, swelling ratio experiments were conducted. Upon application of the hydrogel *in vivo*, the material absorbs body fluids, leading to an increase in volume. This property can enhance the concentration of blood clotting factors and promote the exchange of nutrients and metabolic waste. Subsequently testing of the swelling rates of PG and PG/CB revealed that both reached equilibrium swelling ratio for PG and PG/CB were 229.29 ± 12.6% and 173.95 ± 3.78% respectively (*P* < 0.05) and remained stable for the initial 48 h. The swelling rate of the PG/Van/B/CB system was notably lower than that of the PG hydrogel (Fig. S5a), likely due to two factors: the formation of hydrogen bonds between CB and gelatin chains, as well as electrostatic interactions, which enhance the overall crosslinking degree of the system. Additionally, the CB within the hydrogel system did not exhibit significant swelling behavior, resulting in a higher initial weight of the bone grafts delivery system according to the swelling formula.

The ideal bone graft material must be degradable, allowing new bone tissue to replace the material and promoting bone healing and regeneration. To assess the degradability of the system, the prepared hydrogels were immersed in PBS (0.01 M, pH = 7.4) at a temperature of 37 °C, and their weight changes were subsequently recorded. As illustrated in Fig. S5b, the PG hydrogel was largely degraded within 9–11 days, whereas the degradation time of PG/CB was slightly extended. During the degradation process, the molecular weight of the degradation products was also monitored (Fig. S6 and Table S1). On day one, a distinct low-molecular-weight peak (t = 6.4 min) was observed, corresponding closely to the molecular weight of PEG, indicating the early cleavage of ester bonds between PEG and gelatin. On day two, a high-molecular-weight peak emerged with the Mn of 83,656. Considering the pure gelatin has a Mn of 76,466, the results likely indicated the released gelatin were conjugated with PEG. As time progressed, the area of the high-molecular-weight region increased, indicating the gradual release of more gelatin molecules. Initially, PEG molecules were released from the network, likely due to their bifunctional nature. Subsequently, gelatin molecules were released, which can be attributed to the presence of multiple amino groups on gelatin that could form more crosslinks within the network. As a result, it is concluded the biodegradability of the PG adhesive hydrogel in PBS can be attributed to the presence of ester bonds in its structure.

During the early stages of bone injury, some enzymes which can degrade gelatin are secreted. Therefore, we conducted further *in vitro* degradation experiments in an enzymatic environment. Fig. S7 shows that the presence of gelatinase accelerated the degradation of the PG hydrogel (∼3 days), indicating that the hydrogel can undergo both hydrolysis and enzymatic degradation.

To further demonstrate its degradation behavior, the *in vivo* degradation experiments were performed by ICG-labelling. In the degradation model *in vivo*, the PG hydrogel was present at the defect site of the femoral condyle in SD rats for over 28 days but nearly disappeared at day 42 (Fig. S8), suggesting that a prolonged *in vivo* degradation compared to that of *in vitro*. This may be due to the lower enzyme concentration and the relatively limited fluid at the site of the femoral condyle defect.

The primary role of PG hydrogel when implanted at the bone defect site is to fix the CB at the bone grafting site and provide some mechanical support to maintain the stability of the bone defect area. For bone regeneration, the period from 0 to 2 weeks post-bone injury is the hematoma formation phase, while 3 - 6 weeks is the fibrous callus formation phase. In this study, the *in vivo* degradation test indicates that PG hydrogel covers both the hematoma formation phase and the initial stages of callus formation, meaning that it reduces postoperative displacement and micromotion of bone grafts, enhancing the mechanical stability of the bone defect area during this period. However, for the subsequent ossification phase and modelling phase, which generally occur several months to years after the bone injury, the existence of PG hydrogels would influence the bone regeneration by physically hindering the ingrowth and mineralization of new bone tissue toward the center of the defect. As a result, its degradation will leave more space for new bone regeneration. Meanwhile, CB will play a role in the subsequent stage. CB, as a bone graft material, generally requires 6 months to 2 years for absorption *in vivo*. CB supports the direct mineralization of new bone on its surface, forming a bone integration between the grafted bone and host bone [39]. The material can gradually transfer mechanical loads to the new bone, preventing premature loss of support that could lead to repair failure. Therefore, the designed bone/bioadhesive grafts delivery system here has the potential to be in line with the bone regeneration cycle.

### 3.2 Properties of mechanical properties, self-healing properties, tissue adhesion, injectability, and shape adaptability of the bone grafts delivery system

Generally, the post-mixing of hydrogel system with additional components can influence network integrity due to the breakage of chemical bonds. In our previous research we discovered the self-healing properties of PEG-SG/gelatin-based adhesive hydrogels [29,30]. Inspired by this, we post-mixed CB with the hydrogels to avoid displacement. Experimental testing involving PG and PG/CB hydrogel system in disc-shaped molds confirmed their self-healing properties, even in the presence of CB (Fig. 1d). Because of the self-healing properties of the PG hydrogels, the post-mixture will not hinder the network integrity.

To further demonstrate if the post-mixture would influence the network integrity, mechanical performance of the adhesive hydrogel delivery system was evaluated, before and after self-healing (Fig. 1e). Compression was conducted on PG and PG/CB under varying levels of compression (20%, 40%, and 50%). The results were displayed in Fig. 1f-h and demonstrated a notable increase in compressive strength with the addition of CB, showing an increased cohesion strength of the system. This happens because calf bone, as rigid particles, is embedded in the three-dimensional network of the PG hydrogel. It can directly increase the compression strength by chemically crosslinking with Bi-PEG-SG and restricting the slippage of polymer chains during the compression process. Postoperative bleeding after bone grafting is a major concern, and conventional delivery system typically lack hemostatic properties [2]. In this study, adhesive hydrogels was utilized for bone grafts delivery. The hemostatic performance of the adhesive hydrogel is characterized by its sealing capability, which is most determined by its cohesion strength [25]. The results indicate that the addition of CB can directly enhance the cohesion strength of the adhesive hydrogels, thereby improving its hemostatic effect. Additionally, it reduces micromovements between the bone grafts and the surrounding bone tissue, preventing the bone grafts from breaking or deforming under load, thereby ensuring effective healing and stability of bone defects [40].

In the 20%, 40%, and 50% compression tests, it was observed that the compressive strength of both PG and PG/CB, before and after self-healing, remained almost identical. This indicated that the bone/bioadhesive grafts system possess the capability for mechanical self-healing (Fig. 1f-h). Later, the self-healing properties of the system were also evaluated by assessing changes in mechanical strength through tensile testing. Briefly, two unstained strips were cut at both ends and then reconnected. After incubating at 37 °C for 1 h, Fig. 1i showed that the reconnected strips were connected to each other, which withstood a certain amount of stretching without breaking, proving their self-healing capability. Later, the tensile tests were performed. The results shown in Fig. 1j indicated that both PG/CB and PG exhibited similar tensile strength before and after self-healing, indicating that the addition of CB did not affect the self-healing properties (*P* > 0.05). In the bone/bioadhesive grafts delivery system constructed in this study, the tensile behavior primarily relies on the ductility of the PG hydrogel polymer chains. Therefore, the addition of calf bone does not have a significant impact on the tensile properties. The demonstrated self-healing properties be advantageous in preventing stress concentration in the bone graft material, increasing the material's durability, and effectively transferring loads [41].

Due to the cross-linking characteristics of the self-healing hydrogel network, another advantage is its inherent injectability [42,43], allowing it to be injected via syringe or catheter into deep bone defects during application, facilitating ease of use. Fig. 1k and Video S1 (Supporting Information), demonstrated that the system can be injected through a syringe in the liquids. providing ease of use in clinic. Meanwhile, to further demonstrate injectability, a homemade pneumatic injection system was used to test the injection force of the systems (Fig. S4). At a set airflow rate (0.2 mL/s), an ejection pressure of only ∼13.67 kPa was sufficient to achieve injection. This experiment validates the feasibility of using the homemade pneumatic injection system for *in vitro* injectable delivery of the PG/CB delivery system. Here, to demonstrate the universality of this method in bone grafts delivery, xenogenic bone, autologous bone, and artificial bone also can be injected through a syringe (Video S1-3, Supporting Information), proving the potential of this system to deliver different bone granules.

Supplementary video related to this article can be found at https://doi.org/10.1016/j.bioactmat.2025.07.049

The following is/are the supplementary data related to this article:

Multimedia component 2 Multimedia component 2

Multimedia component 3 Multimedia component 3

Multimedia component 4

Additionally, self-healing hydrogels have excellent adaptability, allowing them to adapt to irregular shapes or different geometries of bone defects through gravity or surface tension. Fig. 1l showed that PG/CB can completely fill an irregular mold, illustrating the fluidity and adaptability of the system. The shape adaptability of bone graft materials allows for uniform distribution in bone defect areas, reducing the formation of dead spaces and enhancing local stability. Altogether, this work introduced a bioadhesive with self-healing properties to deliver bone granules, with good injectability and shape adaptability. Meanwhile, this system has the ability to deliver different kinds of bone graft materials, making it promising in clinic.

Adhesion is very important to sealing ability and prevention of bone grafts displacement. Fig. S9 and Video S4 (Supporting Information) demonstrate that PG/CB remains stable on porcine skin under torsion and water flow, showcasing its adhesiveness. Lap shear tests conducted in accordance with the ASTM standard F2255-05 (Fig. S10a) showed there was no significant difference among the two groups in adhesion strength (PG: 4.98 ± 1.32 kPa; PG/CB: 7.87 ± 3.49 kPa, *P* > 0.05, Fig. S10b). Furthermore, the adhesion to bone was evaluated (Fig. 1m), showing the stable adhesion even under the influence of water flow washing. To further evaluate the adhesion strength of PG/CB delivery system in bone tissue, lap shear tests were performed with bone tissue (Fig. 1n). The results shown in Fig. 1o indicated that both PG and PG/CB exhibited tensile strength without significant differences (*P* > 0.05), demonstrating that the addition of CB dose not significantly influence the adhesion strength.

Supplementary video related to this article can be found at https://doi.org/10.1016/j.bioactmat.2025.07.049

The following is/are the supplementary data related to this article:

Multimedia component 5 Multimedia component 5

The adhesive properties of the bone/bioadhesive system are dictated by both interfacial bonding strength and cohesion strength [44]. The cohesion strength was proved to be enhanced in Fig. 1f-h, contributing to the enhanced adhesive strength. The tissue adhesion can enable the system to adhere to irregular bone defects, thereby reducing the formation of edge gaps and micro-gaps. This minimizes the risk of displacement, micromotion, or collapse of the bone defects, and promotes integration between the bone grafts and the host interface, thereby enhancing the mechanical stability of the entire defect area. Furthermore, the injectability and adaptability of the system facilitate its distribution throughout the defects area, allowing the loaded drug or bioactive molecule to maximize its therapeutic potentials. According to our best knowledge, this is the first paper to fabricate adhesive bone grafts delivery system with self-healing properties.

The bone/bioadhesive system have the ability to deliver different kinds of functional additives, enhancing this system with the ability to exhibit various performance capabilities. To promote bone repair and endow the system with antibacterial properties, Van and rhBMP-2 were also loaded into the system. There are two primary mechanisms responsible for the sustained release. The first one is the diffusion of the agents from the network. The second is the degradation -mediated release. As illustrated in Fig. 1p, the PG/Van/CB system released 19.67 ± 5.6% of Van on the first day and 31.3 ± 3.1% on the second day in PBS (0.01 M, pH = 7.4). Notably, the release rate significantly increased after the fourth day, with 91.67 ± 3.5% released by the 7th day. Van has a relatively low molecular weight (1.45 kDa), and as the hydrogel swells, the pores enlarge, allowing it to diffuse into the surrounding environment through the three-dimensional network pores of PG hydrogel. Based on the classic Higuchi drug release equation Mt/M∞ = kt^1/2^for fitting the release curve (Fig. 1p), the results indicate an R^2^ value of 0.958 (< 0.97), showing that the diffusion is the dominant in Van release [45]. As illustrated in Fig. 1q, the PG/B/CB system demonstrated a release of 4.2 ± 0.59% of rhBMP-2 on the first day, with no significant increase in release observed during the first three days. However, as the system began to degrade, the release rate of rhBMP-2 increased from the fourth day onwards, culminating in a total release of 69.8 ± 4.3% within 15 days. rhBMP-2, being a protein-based bioactive substance with a larger molecular weight (approximately 12.9 kDa), exhibits a significantly slower release rate than vancomycin during the first three days of the release experiment. It is inferred that rhBMP-2 could not easily diffuse and release from the pores of the hydrogel. Meanwhile, the presence of multiple amino groups allows for a stronger covalent attachment to the hydrogel network, limiting the diffusion release. Fitting the release curve according to the classic first-order release kinetics equation Mt = M∞ (1 - e^-kt^) (Fig. 1q) yields an R^2^ value of 0.992 (> 0.97), indicating that the release is accompanied by chemical bond cleavage via the degradation of the hydrogel [46], which mitigated the initial burst release and facilitated sustained release [47]. These findings suggest that the PG/CB system can effectively function as a drug delivery carrier and achieve a substantial release of drugs. In the future, different functional additives can be added for different applications.

### 3.3 Bursting pressure, femoral artery hemostasis in SD rat and hemostasis on femoral condylar defect in New Zealand rabbit

During bone graft surgery, the removal of cortical bone and partially damaged cancellous bone can lead to increased intraoperative bleeding. Excessive bleeding may obscure the surgical field, complicating the procedure. Meanwhile, insufficient hemostasis during surgery necessitates postoperative drainage to prevent hematoma from compressing surrounding soft tissues, significantly increasing the patients’ bedridden time and leading to extra suffering [[48], [49], [50]]. However, none of the reported bone grafts or bone grafts delivery system possess hemostatic properties.

**Fig. 2:**
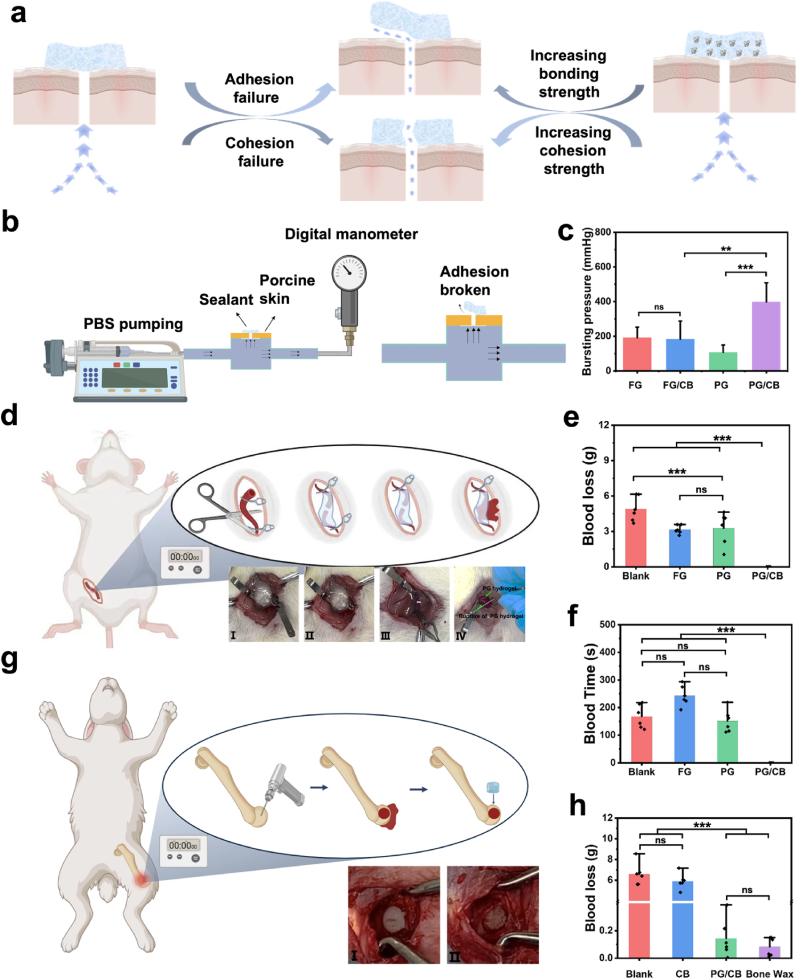
Bursting and hemostasis performance of PG and PG/CB system. a) Scheme shows two ways of bursting after the sealing. The first one is the adhesion failure, in which case increasing the bonding strength will increase the bursting pressure. The second one is the cohesion failure, in which case increasing the cohesion strength will increase the bursting pressure. b) Scheme shows bursting pressure test. c) The result of bursting pressure test. d) The scheme illustrates hemostasis in brachial artery dissection in SD rats. I and II demonstrate the hemostatic effects in the PG/CB group before and after the removal of hemoclips. III showed the hemostatic effects in the PG group before and after the removal of hemoclips and Ⅳ showed the rupture of PG hydrogel in brachial artery dissection of SD rats after the removal of hemoclips. e, f) Blood loss (e) and blood time (f) in brachial artery dissection in SD rats. g) The scheme of hemostasis in femoral defect hemorrhage in New Zealand rabbits. I and II demonstrate the hemostatic effects in the bone wax and PG/CB group. h) Blood loss in 10 min in femoral defect hemorrhage in New Zealand rabbits.

Bioadhesive can promote hemostasis through their strong sealing ability. After sealing, there are two primary mechanisms for leakage (Fig. 2a). The first mechanism involves the breakage of the adhesion area between the sealants and the tissues, which indicates adhesion failure. In this scenario, enhancing the bonding strength will improve the bursting pressure performance. The second mechanism is bulk breakage, which signifies insufficient cohesion strength. In this case, increasing the cohesion strength will lead to higher bursting pressure. Cohesion strength is particularly crucial in sealant manufacturing, as an increase in cohesion strength correlates with higher burst pressure [31]. In our previous work [51], The Bi-PEG-NHS ester, in conjunction with gelatin, was utilized to develop bioadhesive (SEgel) for post-operative anti-adhesion applications. However, the relatively low cohesion strength achieved with the Bi-PEG-NHS ester limits the potential of SEgel in hemostasis.

In this study, the system can be enhanced after the incorporation of CB. One the one hand, the cohesive strength can be enhanced due to the reaction between amino groups carried by CB and Bi-PEG-SG. On the other hand, through the incorporation of micro- and nanoscale CB, the cohesion strength and sealing properties can be enhanced [52]. The improved cohesion strength was demonstrated in the compression test, showing that the mechanical strength of the PG/CB system was significantly greater than that of the PG hydrogels, indicating a marked enhancement in cohesion strength (Fig. 1f-g). As a result, the work introduces a delivery system that delivery vehicle and delivery items can be enhanced by each other.

Subsequently, burst pressure tests were conducted in accordance with ASTM F2392-04 (Fig. 2b). After the incorporation of CB, a significantly increased bursting pressure was observed for the PG/CB system (397.56 ± 110.83 mmHg) compared to PG alone (107.33 ± 42.53 mmHg), and it was superior to commercially available sealants such as fibrin glue (191.89 ± 60.42 mmHg). This clearly demonstrates that the incorporation of CB enhanced the sealing properties, resulting from the increased cohesion strength. However, adding CB to fibrin glue resulted in a burst pressure of only 183.72 ± 104.32 mmHg, indicating no improvement in its sealing properties (Fig. 2c). The incorporation of CB didn't increase the bursting pressure of fibrin glue, which occurred because fibrin glue does not possess self-sealing capabilities, as its structure is compromised during the mixing process. Altogether, the experiments show that the incorporation of CB significantly increased the cohesion strength of the bioadhesive system, making it a perfect system for post-surgery hemostasis.

To demonstrate the *in vivo* hemostatic capability of the system, the femoral artery transection bleeding model was employed in SD rats. In the experiment, after transecting the femoral artery of the SD rats, the artery was clamped using hemoclips, and various treatments were subsequently applied (Fig. 2d). Then, the bleeding time and blood loss were recorded. In the absence of any treatment following the remove of the hemoclips (Blank group), the blood loss was 4.89 ± 1.05 g, and the bleeding time was 167.67 ± 42.60 s, even the death of SD rat occurred (one in six) in the blank group during the experiment. Following the application of fibrin glue and PG hydrogels, blood loss was significantly reduced compared to that of the blank group. The fibrin glue group recorded a blood loss of 3.16 ± 0.37 g, while the PG hydrogels group exhibited a blood loss of 3.28 ± 1.39 g (*P* < 0.001). The bleeding time in the PG hydrogels group was 151.5 ± 41.08 s, which was comparable to that of the blank group (*P* = 0.885). In contrast, the bleeding time in the fibrin glue group was significantly prolonged at 242.83 ± 36.83 s compared to the blank group (*P* < 0.001), as is shown in Fig. 2e and f. After applying fibrin glue (Video S5, Supporting Information) and PG hydrogels (Video S6, Supporting Information), although a decreased blood loss was observed, the bleeding time was still a bit long, showing the limited hemostatic properties. Although the burst pressure of fibrin glue is higher than that of the PG hydrogel, its cohesive strength remains insufficient to effectively stop bleeding from a femoral artery transection. As a result, once the hemoclips was removed, the inadequate cohesive strength led to rupture and rebleeding just as that happened in PG groups. Moreover, thrombin primarily functions during the coagulation phase, and when sealing fails, the hemostatic effects of fibrinogen and thrombin become limited. Consequently, in the SD rat femoral artery transection hemorrhage model, both the blood loss and the bleeding time are comparable to those observed with PG hydrogel. Notably, in the PG/CB group, no bleeding was observed at the transected end after the hemoclips were removed (Fig. 2d II and Video S7, Supporting Information), with both bleeding time and blood loss recorded as zero, showing the good sealing abilities of the PG/CB system. This happened because of the increased cohesion strength after the addition of CB, enhancing the sealing ability and leading to leakage sealing of bleeding after application [25]. During the bleeding experiments, it was noted that the PG group exhibited cohesion rupture bleeding (Fig. 2d IV and Video S6, Supporting Information), while nothing similar occurred in the PG/CB group (Fig. 2d II and Video S7, Supporting Information), further confirming that the increased cohesion strength was the reason for the increased sealing ability.

Supplementary video related to this article can be found at https://doi.org/10.1016/j.bioactmat.2025.07.049

The following is/are the supplementary data related to this article:

Multimedia component 6 Multimedia component 6

Multimedia component 7 Multimedia component 7

Multimedia component 8

To further evaluate the bone hemostatic effect of PG/CB in bone defects area, a femoral condyle defect hemostasis model was established in New Zealand rabbits (Fig. 2g). Using an orthopedic hand drill, a cylindrical defect with a diameter and height of 6 mm was created on the lateral femoral condyle, with the bleeding loss recorded over a 10-min period. Bone wax, recognized for its excellent hemostatic properties, ease of use, and low cost, has served as a classic hemostatic material in orthopedic surgery for nearly a century [53]. As illustrated in Fig. 2h, simple implantation of CB resulted in a bleeding volume comparable to that of the untreated group (blank group: 6.69 ± 1.08 g; CB group: 5.91 ± 0.76 g), indicating a lack of hemostatic effect (Video S8, Supporting Information). This result aligns well with the clinical situations that bleeding was still observable around the bone tissue after the use of bone grafts [17]. In contrast, the bleeding loss in the PG/CB groups were significantly lower (0.14 ± 0.08 g, Fig. 2h), demonstrating excellent hemostatic performance (Fig. 2g III and Video S9, Supporting Information), showing similar hemostatic performance compared to that of bone wax (Fig. 2g I and Video S10, Supporting Information). Although bone wax has served as a classic hemostatic material for treating bone bleeding in orthopedic surgery for nearly a century, its low biodegradability adversely impacts bone regeneration [17]. Meanwhile, clinical case reports suggest that the use of bone wax may pose risks of allergic reactions and foreign body rejection at the surgical site [54,55]. Despite these concerns, bone wax remains widely utilized in orthopedic surgery due to the absence of effective alternative. In this study, the PG/CB system exhibited hemostatic performance comparable to that of bone wax. Meanwhile, the injectable, degradable, and shape-adaptable PG/CB adhesive hydrogel system positioning it as an ideal alternative for bone hemostatic materials.

Supplementary video related to this article can be found at https://doi.org/10.1016/j.bioactmat.2025.07.049

The following is/are the supplementary data related to this article:

Multimedia component 9 Multimedia component 9

Multimedia component 10 Multimedia component 10

Multimedia component 11 Multimedia component 11

### 3.4 Antioxidant of bone/bioadhesive grafts delivery system

Lines of evidence have shown that hypoxia-induced overproduction of reactive oxygen species (ROS) in the bone defects region significantly impacts the delay of bone regeneration [56]. Consequently, being antioxidant during the bone repair process is essential for facilitating normal bone healing and ensuring functional recovery. Gelatin, a hydrolysate of collagen, has been reported to possess antioxidant properties [57,58]. Unlike potentially harmful synthetic antioxidants, gelatin is FDA-approved and exhibits clear biocompatibility [38].

**Fig. 3:**
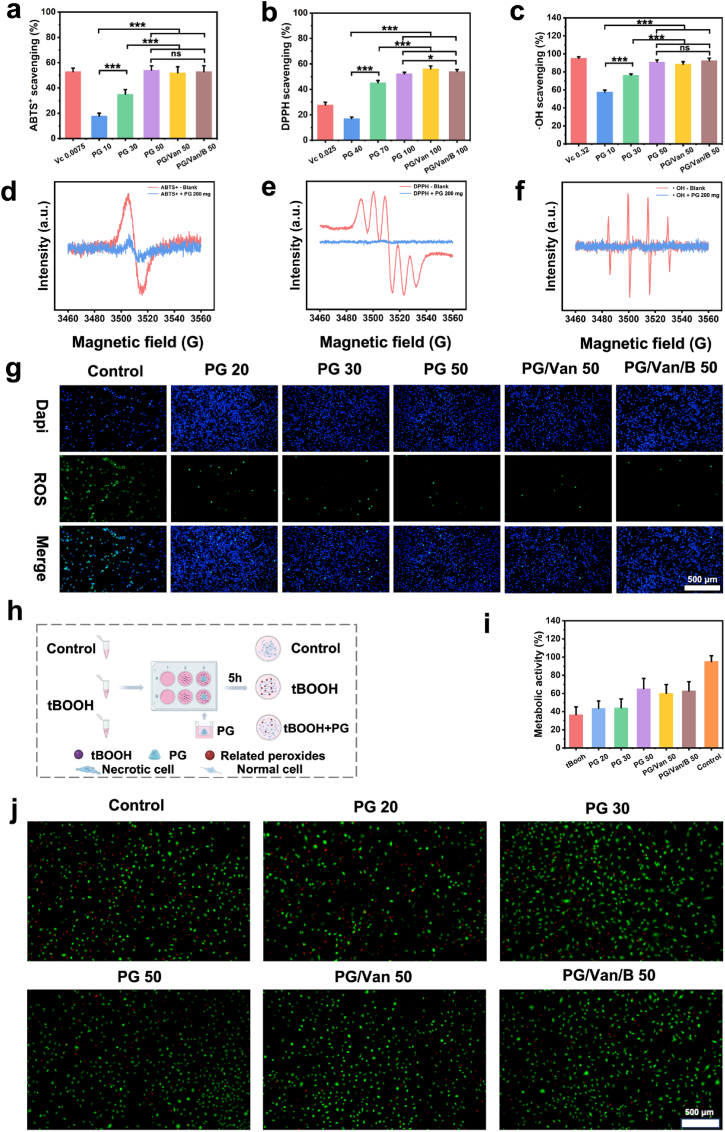
PG/Van/B efficiently scavenges free radicals both in *vitro* and in cell culture. a, b, c) Results showing the scavenging rate of ABTS+⋅ (a), DPPH (b), and ·OH (c), compared with ascorbic acid (Vc) solutions. d, e, f) The scavenging of ABTS+ (d), DPPH (e) and ·OH (f) as characterized by EPR. g) The scavenging of intracellular ROS results of NIH 3T3 cells when incubated with PG hydrogels of different weight and different functional additives. (Scale bar = 500 μm). h) Scheme showing antioxidant cell model. The oxidative stress was induced by adding tert-butyl hydroperoxide solution (tBOOH). As control, the tBOOH and different weight of PG and PG/Van/B were added together to show the hydrogels whether or not rescue cells from oxidative stress caused by tBOOH. i) Metabolic activity of NIH 3T3 cells. j), Live/dead cell staining results of NIH 3T3 cells when incubated with tBOOH alone and tBOOH with PG hydrogels of different weight and loading different functional additives. (Scale bar = 500 μm, n = 6, mean ± SD).

In this study, it was hypothesized that the system would demonstrate antioxidant properties due to its gelatin-based composition. The system's ability of PG/Van/B to scavenge various free radicals, including ABTS+, DPPH, and ·OH, was evaluated. Fig. 3a and b and c illustrated that the scavenging activity of the bioadhesive against different types of free radicals is dose-dependent, indicating robust antioxidant properties. The free radical scavenging properties were further evaluated by EPR. As shown in Fig. 3d-f, complete scavenging of DPPH and ·OH was achieved, and most of the ABTS+ was also scavenged. This indicates that the PG hydrogel has the ability to scavenge free radicals. To further investigate the systems' ability to scavenge intracellular ROS free radicals, the experiment of scavenging of intracellular ROS free radical was performed (Fig. 3g). It was showed that all PG hydrogels at chosen concentrations reduced the production of ROS free radical with less green fluorescence, and the scavenging ability increases with dosage. Notably, the loading of van and rhBMP-2 does not affect the inherent antioxidant activity of the PG hydrogel. In summary, our experiments demonstrate that the PG hydrogel has the ability to scavenge free radicals. Consequently, the bioadhesive have the potential to mitigate tissue damage caused by oxidative stress by effectively quenching free radicals associated with this condition, thereby reducing fibrosis and promoting tissue healing. Additionally, an oxidative stress cell model was established to assess whether bioadhesive could rescue cells. As shown in Fig. 3h, tBOOH was utilized to induce oxidative stress in cells, resulting in apoptosis. Following the introduction of tBOOH, the metabolic activity of NIH 3T3 cells significantly decreased to 38.6 ± 8.9% (Fig. 3i). However, upon the addition the bioadhesive, the metabolic activity nearly doubled, increasing to approximately 60%. This enhancement in metabolic activity indicated that the bioadhesive can aids cells in evading oxidative stress. This finding was further corroborated by a live/dead cell staining kit, which revealed a higher number of live cells and fewer dead cells in the PG/Van/B treatment group (Fig. 3j). In summary, the above experiments demonstrate the antioxidant properties of PG hydrogels, which can help modulate the free radicals in bone defect areas, protecting osteoblasts, endothelial cells, and other cells from oxidative damage, and promoting bone regeneration.

### 3.5 Biocompatibility of bone/bioadhesive grafts delivery system

**Fig. 4:**
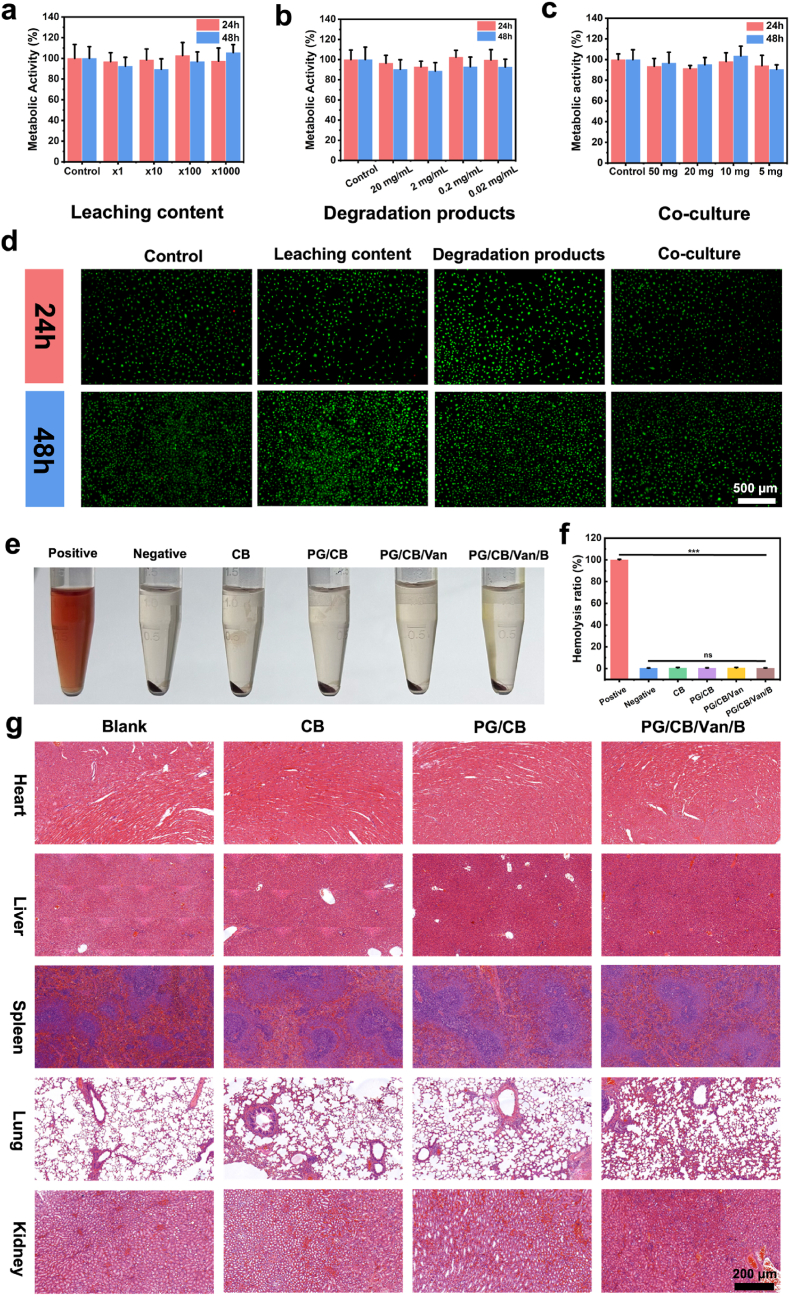
PG/Van/B/CB showed promising biocompatibility *in vitro* and *vivo*. a, b, c) Metabolic activity of NIH 3T3 cells when incubated with leaching content (a), degradation products of PG/Van/B/CB (a), and co-cultured with PG/Van/B/CB (a). d) Live/dead cell staining of NIH 3T3 when incubated with leaching content, degradation products of PG/Van/B/CB and co-cultured with PG/Van/B/CB (Scale bar = 500 μm). e, f) Showing the hemolysis rate of CB, PG/CB, PG/CB/Van, PG/CB/Van/B (n = 3, mean ± SD, ns: not significant, ∗∗∗*P* < 0.001). g) The CB, PG/Van/CB, PG/Van/B/CB were placed in the left dorsum of the rats (n = 3). After 4 weeks, the rats were euthanized by cervical dislocation. Critical organs such as the heart, liver, spleen, lungs, and kidneys were harvested for H&E staining (Scale bar = 200 μm).

According to FDA and other regional regulations, the safety and efficacy of biomaterials are equally important [60]. In this study, both PEG and gelatin are recognized as FDA-approved biomaterials, ensuring the biocompatibility of the resulting system. To verify the biocompatibility of this system, the CCK-8 assay was employed to evaluate the compatibility of the system, the leaching and degradation products of the system. Fig. 4a and b and c demonstrated that at the selected concentrations, the metabolic activity of all samples reached 80%, indicating that PG/Van/B/CB possesses good biocompatibility [61]. Live/dead cell staining (Fig. 4d) revealed a minimal presence of dead cells, further corroborating this finding and illustrating that PG/Van/B/CB also exhibits strong biocompatibility. Given that the system will contact wounds and be exposed to blood, hemocompatibility was assessed by incubating the system with saline containing 2% (v/v) red blood cells. Fig. 4e, f indicated that at the selected concentrations, the hemolysis rate across all sample groups was below 5%, signifying good hemocompatibility. To further investigate whether PG/Van/B/CB induces systemic toxicity *in vivo*, the system was subcutaneously implanted in SD rats for 4 weeks. Following this period, the rats were euthanized, and major organs were subjected to H&E staining. The H&E-stained images of the major organs (heart, liver, spleen, lungs, and kidneys, Fig. 4g) revealed no histological abnormalities after the system implantation, indicating good biosafety. The good biosafety further enhances the translational potential of the bone/bioadhesive delivery system. In summary, this is an effective fabricating method that can safely deliver various bone granules without raising any safety concerns

### 3.6 Bacteriostasis of bone/bioadhesive grafts delivery system

Infection not only impairs bone healing but can also lead to severe complications, such as osteomyelitis [62]. As a result, it is preferred to have antibacterial properties in the system. Another function of this delivery system is that it can easily load different functional additives. Here, Van was chosen to achieve the antibacterial property, which is widely used post-operatively in orthopedics due to its potent bactericidal effect against *Staphylococcus aureus* (*S.aureus*) [63].

**Fig. 5:**
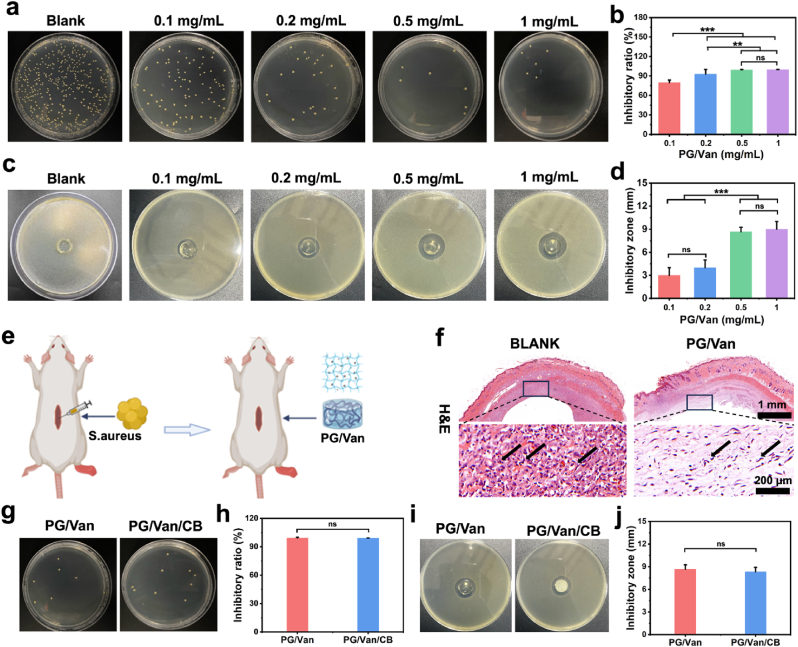
Antibacterial property of PG/Van and PG/Van/CB *in* vitro and in viv*o*. a, b) Images show that the antibacterial ratio of PG/Van increases with rising Van concentrations (a), as confirmed by quantitative analysis of the antibacterial ratio (b). c, d) Images show that inhibition zone diameter of PG/Van increases with rising Van concentrations (c) and quantitative analysis of inhibition zone diameter (d). e) Scheme showing antibacterial *in vivo*. *S.aureus* was injected into the subcutaneous tissue of SD rats. PG/Van was then embedded at the site of *S.aureus* injection. The incision was sutured and co-cultured for 7 days before being examined. Subcutaneous tissue samples were taken for H&E staining to observe the infiltration of inflammatory cells. f) H&E staining images around subcutaneous tissue embedded with or without hydrogels at 7 days post-operation (Scale bar = 1 mm (top row), Scale bar = 50 μm (bottom row)). g, h) Pictures show that the antibacterial ratio at 0.5 mg/mL (g) of Van in PG/Van and PG/Van/CB and quantitative analysis (h). i, j) Pictures show that the inhibition zone diameter at 0.5 mg/mL of Van in PG/Van and PG/Van/CB (i) and quantitative analysis (j).

To assess the *in vitro* antibacterial effect of the PG hydrogels, a suspension of *S.aureus* (10^6^ CFU/mL) was co-incubated with PG/Van formulations containing different concentrations of Van for 12 h. All the groups containing Van showed antibacterial effects (Fig. 5a and b). The antibacterial efficacy increased initially with the increase of the Van concentrations. However, when the Van concentration was increased from 0.5 mg/mL to 1 mg/mL, the inhibition rate showed no significant, indicating that the PG/Van concentration of 0.5 mg/mL exhibits strong antibacterial activity. Further, the inhibition zone experiments were performed to demonstrate the system's antibacterial properties. After co-incubating PG hydrogels loaded with varying concentrations of Van with *S.aureus* for 12 h, a distinct inhibition zone was observed on the agar plates (Fig. 5c). The diameter of the inhibition zone increased with higher Van concentrations (Fig. 5d). At the Van concentration of 0.5 mg/mL, the inhibition zone diameter was measured 8.67 ± 0.58 mm. When the Van concentration was increased to 1 mg/mL, the inhibition zone diameter increased to 9 ± 1 mm. This phenomenon was the same as the coincubation experiments. As a result, subsequent experiments employed PG/Van with the Van concentration of 0.5 mg/mL.

To evaluate the antibacterial activity of PG/Van, a 7-day subcutaneous incubation of *S.aureus* with PG/Van in SD rats was performed (Fig. 5e), followed by H&E staining to assess the soft tissue inflammatory response (Fig. 5f). The control group exhibited substantial accumulation of lymphocytes, monocytes, and neutrophils subcutaneously, indicating a pronounced inflammatory response. In contrast, the PG/Van group demonstrated a significant reduction in inflammatory cell infiltration, suggesting that PG/Van effectively targets Gram-positive *S.aureus* and mitigates local inflammation [59]. These findings indicate considerable potential for PG/Van in protecting open wounds and preventing infections.

The antibacterial properties of the bone/bioadhesive system (PG/Van/CB, Van: 0.5 mg/mL) after loading CB were also further investigated, and Fig. 5g-j demonstrated that the addition of CB did not impact the antibacterial effectiveness. Overall, due to the good delivery abilities of the adhesive hydrogels, the systems can load antibiotics for infection prevention.

### 3.7 Angiogenic and osteogenic capacity *in vitro* of bone/bioadhesive grafts delivery system

When using autografts, allografts, xenografts, or certain biomaterials for the repair of critical bone defects or non-healing fractures, the speed and efficiency of the repair become critical concerns [64]. Calf bone is the most widely used bone substitute material globally [65]. However, it possesses relatively poor osteoinductive properties. As a result, it is preferred to have bioactive factors for bone regeneration in the bone/bioadhesive grafts delivery system. Here, rhBMP-2 was chosen to achieve the better bone regeneration effect, which can induces undifferentiated mesenchymal stem cells to differentiate into new bone tissue [66].

In this study, to investigate the *in vitro* angiogenic capacity of the system, scratch assays, transwell assays, and matrigel tube formation assays were conducted using Human umbilical vein endothelial cells (HUVECs). To maintain the effective concentration of rhBMP-2 released from the system into the culture medium during the *in vitro* induction process, the PG/B/CB were prepared and soaked in the culture medium at 4 °C to facilitate rhBMP-2 release [67]. Then the leaching solutions were used to culture HUVECs.

**Fig. 6:**
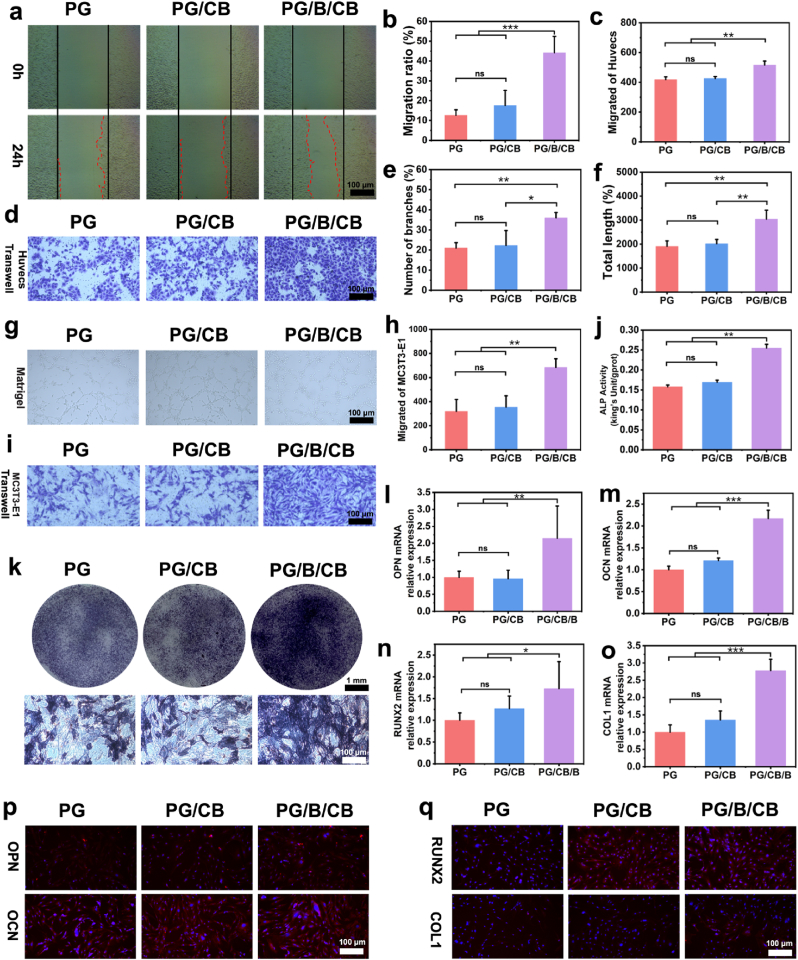
a) Scratch assay results: The area between the black solid lines and the red dashed lines is the scratch wound healing. b) Quantitative analysis evaluating the migration activity of HUVECs cultured with the leaching content of CB, PG/CB PG/B/CB (Scale bar = 100 μm). c, d) Transwell assay accessing the migration activity of HUVECs cultured with the leaching content of CB, PG/CB, PG/B/CB where the migrated cells were stained with crystal violet, and quantitative analysis of the number of migrated HUVECs using ImageJ software (Scale bar = 100 μm). e, f, g) Optical microscope images of matrigel experiment evaluating the tube formation ability of HUVECs cultured with the leaching content of CB, PG/CB, PG/B/CB, and quantitative analysis of the number of branches and total length formed by HUVECs using ImageJ software (Scale bar = 100 μm). h, i) Transwell assay accessing the migration activity of MC3T3-E1 cultured with the leaching content of CB, PG/CB, PG/B/CB where the migrated cells were stained with crystal violet, and quantitative analysis of the number of migrated MC3T3-E1 using ImageJ software (Scale bar = 100 μm). j, k) Alkaline phosphatase staining of MC3T3-E1 cultured with the leaching content of CB, PG/CB, PG/B/CB and alkaline phosphatase activity assay (Scale bar = 1 mm (top row), Scale bar = 100 μm (bottom row)). l - o) Osteogenic gene (OPN, OCN, RunX2 and COL1) expression of cultured with MC3T3-E1 cultured with the leaching content of CB, PG/CB, PG/B/CB evaluated by qPCR. p, q) Immunofluorescence of osteogenic proteins (OPN, OCN, RunX2 and COL1) expression of cultured with MC3T3-E1 cultured with the leaching content of CB, PG/CB, PG/B/CB (Scale bar = 100 μm).

First of all, the scratch assays were performed to show the ability of the systems to promote HUVECs migration. As shown in Fig. 6a, identical scratches were created in each culture well at the start (0 h). After 24 h of healing, the PG and PG/CB groups demonstrated minimal healing, whereas the PG/B/CB group exhibited significant healing as indicated between blank solid lines and the red dashed lines, markedly enhancing cell migration. Further, quantitative analysis indicated that the HUVECs migration rate in the PG/CB/B group was 2.51 times greater than that in the PG/CB group (Fig. 6b). The scratch assays demonstrated that the addition of rhBMP-2 significantly accelerate the cell migration of HUVECs, which may be good for angiogenesis.

Then, transwell assay utilizing crystal violet staining was conducted to characterize cell penetration, thereby confirming the system's ability to recruit cells. HUVECs in the PG/B/CB group demonstrated significantly greater cell migration compared to the PG and PG/CB group. Further quantitative analysis revealed that the migration of HUVECs in the PG/B/CB group was 1.22 times higher than in the PG/CB group (Fig. 6c and d). This experiment validated the system's ability to recruit HUVECs, confirming that the addition of rhBMP-2 endowed the system with remarkable angiogenic cell recruitment.

To further illustrate the angiogenic properties of the system, a Matrigel tube formation assay-directly reflecting the level of angiogenesis-was performed. As depicted in Fig. 6e and g. The PG/B/CB group formed a greater number of tubular structures. Additional quantitative results indicated that the number of branches and the total length in the PG/B/CB group increased by 1.6 and 1.51 times, respectively, compared to the PG/CB group. In summary, the PG/B/CB system effectively accelerates the HUVECs cell migration, enhance their recruitment, thereby improving the angiogenic performance of the composite hydrogel, which are consistent with the reports on the pro-angiogenic effects of rhBMP-2 [[68], [69], [70]].

After evaluating the system's performance on angiogenesis, the osteogenic experiments were performed using MC3T3-E1 cells. First of all, the transwell assay was conducted to characterize cell penetration, thereby confirming the system's effect on cell recruitment. MC3T3-E1 cells in the PG/B/CB group demonstrated significantly greater cell migration compared to the PG/CB group. Further quantitative analysis revealed that the migration of MC3T3-E1 cells in the PG/B/CB group was 1.93 times higher, than in the PG/CB group (Fig. 6h and i). This experiment validates the system's ability to recruit MC3T3-E1 cells, which is helpful for bone generation.

Later, to show osteogenic capacity of bone/bioadhesive grafts delivery system, MC3T3-E1 cells after co-cultured with extracts of PG, PG/CB, and PG/B/CB underwent alkaline phosphatase (ALP) staining. After 14 days of co-culture, ALP staining and activity measurements were performed. The color intensity observed in the PG and PG/CB groups was significantly weaker compared to the PG/B/CB group (Fig. 6k). Subsequent ALP activity measurements indicated no significant between the PG and PG/CB groups. However, both exhibited significantly lower activity than the PG/B/CB group (*P* < 0.05, Fig. 6j).Furthermore, q-PCR was employed to assess the gene expression of Osteopontin (OPN), Osteocalcin (OCN), Runt-related transcription factor 2 (RUNX2) and collagen type I (col1) as is shown in Fig. 6l-o. OPN, OCN, RUNX2 and Col1 were chosen because they are critical markers associated with osteoblast differentiation [71]. After 14 days of incubation, the PG/B/CB group demonstrated the highest levels of gene expression for OPN, OCN, RUNX2, and Col1, significantly surpassing those of the PG and PG/CB groups. This indicates that the rhBMP-2 released in the PG/B/CB group more effectively induced the osteogenic differentiation of MC3T3-E1 cells. 10.13039/100014337Furthermore, the immunofluorescence staining of OPN, OCN, RUNX2 and Col1 was conducted after 14 days of incubation (Fig. 6p, q and S11, Supporting Information). The fluorescence intensity in the PG/CB/B group was higher than that in the PG and PG/CB groups. The increased fluorescence intensity illustrates that MC3T3-E1 cell are undergoing a transition from an undifferentiated state to an osteoblastic lineage. In summary, the incorporation of rhBMP-2 into PG/CB delivery system creates a more favorable osteogenic microenvironment, thereby promoting both the recruitment and osteogenic differentiation of osteoblasts.

### 3.8 Angiogenic and osteogenic capacity *in vivo* of bone/bioadhesive grafts delivery system

**Fig. 7:**
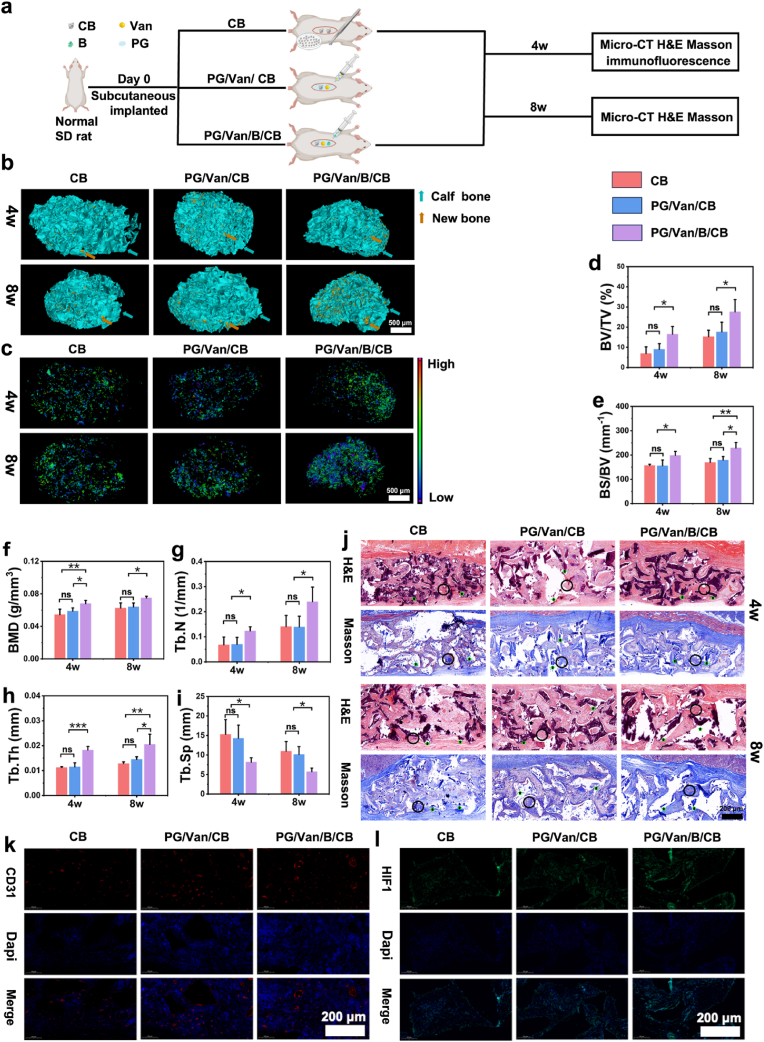
Micro-CT analysis of new bone formation of subcutaneous heterotopic osteogenesis at 4 weeks and 8 weeks and histological analysis. a) Scheme showing the establishment of the bone defect and the timeline of animal experiments to test angiogenic capacity in SD rat of subcutaneous (Scale bar = 500 μm). b) Reconstructed 3D patterns from micro-CT images of subcutaneous heterotopic osteogenesis at 4 weeks and 8 weeks. new bone is represented in yellow and calf bone is represented in green. c) the new bone is reconstructed into different colors according to the CT gray value (tissue Scale bar = 500 μm). d-i) Micro-architectural parameters of the new bone, including BMD, BV/TV, BS/BV, Tb.Th, Tb.Sp, Tb.N. j) Histological evaluation of new bone at 4 weeks and 8 weeks, new bone is marked in black circle; calf bone is marked in green arrow (Scale bar = 200 μm). k, l) Immunofluorescence of angiogenic proteins CD31 and HIF1 at 4 weeks. Date was analyzed via a one-way ANOVA and are shown as the mean ± SD (∗*P* < 0.05, ∗∗*P* < 0.01, ∗∗∗*P* < 0.001, n = 3).

To investigate potential application of the system in bone regeneration, subcutaneous ectopic osteogenesis experiments were first conducted in SD rats to verify osteogenic capacity (Fig. 7a). CB, PG/Van/CB, and PG/Van/B/CB were implanted into the subcutaneous tissues on the back of the SD rats. After 4 and 8 weeks postoperatively, the subcutaneous tissues were examined using Micro-CT to assess the quality and structural characteristics of bones by evaluating different parameters, including bone mineral density (BMD, an indicator of bone strength), bone volume fraction (BV/TV, an indicator for evaluating bone volume to total volume and reflecting the proportion of bone tissue in the region of interest), bone surface area to bone volume ratio (BS/BV, an indicator of the complexity and porosity of the bone surface), trabecular number (Tb.N, an indicator of the number of trabecular within a given volume), trabecular thickness (Tb.Th, an indicator of the average thickness of trabecular, serving as an important indicator of trabecular strength.), and trabecular separation (Tb.Sp, an indicator of the degree of trabecular spacing).

Here, new bone is represented in yellow, while CB is shown in green (Fig. 7b) indicating that there was a minimal amount of new bone in the CB groups. However, when CB was incorporated into the PG hydrogel (PG/Van/CB group), an increase in the amount of new bone was observed. This clearly demonstrates that the PG hydrogel significantly enhanced the bone regeneration outcomes of the CB, owing to increased bone fixation and antioxidant effects of the PG hydrogel on the bone grafts material. Among all the groups, PG/Van/B/CB group displayed relatively the most and densest new bone tissue indicating that the addition of rhBMP-2 accelerated new bone regeneration. To further illustrate the state of bone regeneration, new bone is color-coded based on CT gray values in Fig. 7c. A similar phenomenon was observed, revealing that the PG/Van/B/CB group contained the largest amount of new bone. Meanwhile, as the duration of implantation increased, the quantity of new bone growth in each group also increased (Fig. 7c). In summary, the Micro-CT experiments demonstrate that PG/Van/B/CB accelerates bone regeneration, likely due to the effects of bone fixation, antioxidant properties, and the functional additives incorporated.

To further demonstrate the therapeutic outcome, quantitative analysis from Micro-CT was performed. Fig. 7d-h indicated that the average value of the amount of newly formed bone in the PG/Van/CB group was slightly better compared to the CB group, and the PG/Van/B/CB group exhibited largest values for BMD (0.068 ± 0.004 and 0.075 ± 0.002 g/cm^3^ at 4 and 8 weeks, respectively), BV/TV (16.51 ± 3.8% and 27.62 ± 6.1% at 4 and 8 weeks, respectively), BS/BV (198.34 ± 16.44 and 228.96 ± 22.31 at 4 and 8 weeks, respectively), Tb.N (0.12 ± 0.016 and 0.21 ± 0.004 1/mm at 4 and 8 weeks, respectively), and Tb.Th (0.018 ± 0.002 and 0.021 ± 0.004 mm at 4 and 8 weeks, respectively) compared with the other groups (Fig. 7d-h), indicating that the rhBMP-loaded bone/bioadhesive grafts delivery system could enhance the quality and structural properties of new bone. Furthermore, it displayed significantly lower values for Tb.Sp (8.17 ± 1.12 and 5.74 ± 0.91 mm at 4 and 8 weeks, respectively) compared to the CB and PG/Van/CB groups (Fig. 7i), indicating that the system could improve the trabecular sparing of the new bone in rats. Overall, the results showed that the addition of rhBMP-2 compensated for the insufficient osteoinductive activity of PG/Van/CB, enhanced the quantity and quality of new bone, and promoted bone regeneration.

To further verify the bone regeneration effects across the different groups, H&E and Masson stainings were performed to evaluate bone regeneration. At 4 weeks post-surgery, the CB and PG/Van/CB groups exhibited more connective tissue and less new bone formation in the bone defects area. In contrast, clear evidence of considerable formation of new bone forming dense and organized tissue along the CB in the PG/Van/B/CB group were observed (new bone is represented in black circle and CB is represented in green arrow, Fig. 7j). Meanwhile, at 8 weeks post-surgery, the PG/Van/B/CB group demonstrated higher collagen deposition and stronger collagen matrix staining intensity compared to the CB and PG/Van/CB groups, which was consistent with the H&E results, indicating that the PG/Van/B/CB group enhanced new bone formation.

Angiogenesis is a crucial prerequisite for the maturation of bone regeneration, and there exists a close relationship between angiogenesis and bone regeneration. Particularly in the absence of bone progenitor cells from the periosteum or bone marrow, angiogenesis is essential for achieving functional bone regeneration in the subcutaneous environment. It may facilitate the transfer of bone progenitor cells to the defects site and enhance the transport of oxygen, nutrients, and the clearance of metabolic waste. The immunofluorescence results indicated enhanced expression of CD31 and HIF1 (Fig. 7k-l and Figs. S11–12), suggesting that the addition of rhBMP-2 promoted cell recruitment and angiogenic properties. In summary, the PG/Van/B/CB group exhibits the most effective bone regeneration, attributing to the addition of rhBMP-2. In future studies, additional or different functional additives can be further added to improve the bone regeneration or be suitable for different clinic situations.

**Fig. 8:**
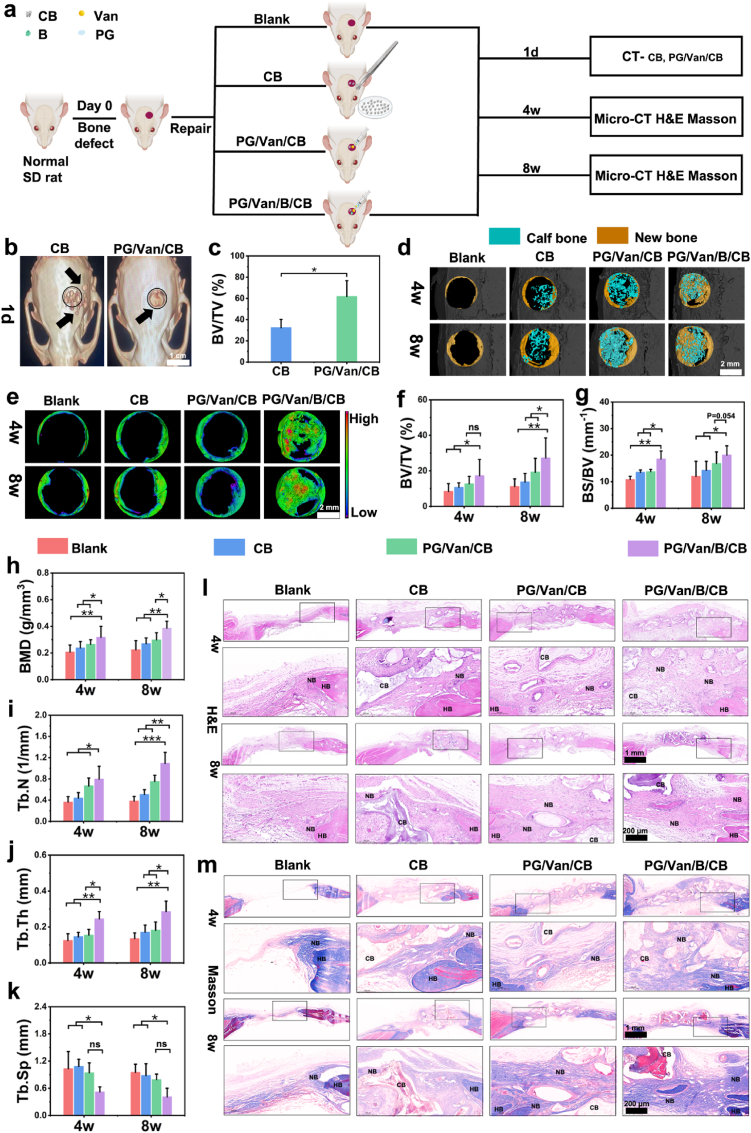
Micro-CT analysis of new bone formation of cranial defect model of SD rats and histological analysis. a) Scheme showing the establishment of the bone defect and the timeline of animal experiments to test therapeutic effect. b) the position of the grafts in the CB and PG/Van/CB groups one day after surgery using CT and 3D reconstruction (Scale bar = 1 mm). c) Micro-architectural parameters (BV/TV) of the CB in the CB and PG/Van/CB groups 4 weeks after surgery using Micro-CT. d) Reconstructed 3D patterns from micro-CT images of osteogenesis at 4 weeks and 8 weeks. new bone is represented in yellow and CB is represented in green (Scale bar = 2 mm). e) the new bone is reconstructed into different colors according to the CT gray value (Scale bar = 2 mm). f-k) Micro-architectural parameters of the newly formed bone: BMD, BV/TV, BS/BV, Tb.Th, Tb.Sp, Tb.N. l, m) Histological evaluation of newly formed bone at 4 weeks and 8 weeks, CB-calf bone; NB-new bone; HB-host bone. Data were analyzed via a one-way ANOVA and are shown as the mean ± SD (∗*P* < 0.05, ∗∗*P* < 0.01, ∗∗∗*P* < 0.001, n = 3).

To further investigate the potential application of PG/Van/B/CB in bone defects repair, the bone regeneration experiment was performed on the SD rat skull defect model (Fig. 8a). After creating a 5 mm bone defect, CB, PG/Van/CB, and PG/Van/B/CB were implanted into the skull defect sites. The retention stability of CB was evaluated first by CT, after 1 day of implantation. The CT image illustrated that when using CB alone for defect repair, significant displacement of the CB occurred (marked by black arrow in Fig. 8b), which may happen due to muscle tension and suturing following surgery. In contrast, in the bioadhesive group, the transplanted CB is located at the bone defect site, demonstrating that the bioadhesive can provide adhesion and fix CB at the site of the bone defect. This stable fixation will improve the therapeutic efficacy of the system. Quantitative analysis of the CB at the bone defect site at 4 weeks showed that the PG/Van/CB group had a greater BV/TV value (Fig. 8c), further confirming that transplanted CB could indeed be fixed at the bone defect.

Furthermore, Micro - CT scans were performed at 4 weeks and 8 weeks post-surgery to assess the repair status of the bone defect. Here, new bone is represented in yellow, while CB is shown in green (Fig. 8d), indicated that there was a minimal amount of new bone in both the Blank and CB groups. However, when CB was incorporated into the PG hydrogels (PG/Van/CB group), a significant increase in the amount of new bone was observed. This clearly demonstrated that the PG hydrogels enhanced the therapeutic outcomes of the CB, owing to the bone adhesion and antioxidant effects of the PG hydrogel on the bone grafts material. Among all the groups, PG/Van/B/CB exhibited the best performance, indicating that the addition of rhBMP-2 accelerated new bone regeneration. To further illustrate the state of bone regeneration, new bone is color-coded based on CT gray values in Fig. 8e. A similar phenomenon was observed, revealing that the PG/Van/B/CB group contained the largest amount of new bone. In summary, the Micro-CT experiments demonstrates that PG/Van/B/CB accelerates bone regeneration, likely due to the effects of bone adhesion, antioxidant properties, and the functional additives incorporated.

Further, quantitative analysis from Micro-CT was performed. Fig. 8f-j indicated that the PG/Van/B/CB group exhibited largest values of BMD (0.387 ± 0.083 and 0.436 ± 0.052 g/cm^3^ at 4 and 8 weeks, respectively), BV/TV (22.26 ± 9.16% and 34.23 ± 11.28% at 4 and 8 weeks, respectively), BS/BV (23.54 ± 5.99 and 28.02 ± 7.45 at 4 and 8 weeks, respectively), Tb.N (0.796 ± 0.241 and 0.991 ± 0.207 1/mm at 4 and 8 weeks, respectively), and Tb.Th (0.245 ± 0.041 and 0.286 ± 0.058 mm at 4 and 8 weeks, respectively) among all the groups. This indicated that the rhBMP-loaded bone/bioadhesive grafts delivery system could enhance the quality and structural properties of new bone. Additionally, it had significantly lower values of Tb.Sp (0.52 ± 0.11 and 0.41 ± 0.19 mm at 4 and 8 weeks, respectively) compared to the Blank, CB, and PG/Van/CB groups, indicating that the system could improve the trabecular sparing of the new bone(Fig. 8k). In summary, the PG/Van/B/CB bone/bioadhesive graft delivery system not only accelerates bone regeneration but also enhances the quality and structural properties of bone by improving the retention of CB and boosting the overall bone inductive activity of the system though rhBMP-2.

Finally, H&E and Masson stainings were performed to evaluate the bone regeneration effects among different groups (CB-calf bone, NB-new bone, HB-host bone, Fig. 8l and m). In the blank group at 4 and 8 weeks, the bone structure surrounding the defect area was discontinuous, with only partial new bone formation observed. The CB group demonstrated minimal bone tissue formation at the margins of the bone defect and between CB particles, exhibiting limited new bone formation and poor structural function. This indicated that the displacement of bone graft materials was unfavorable for bone growth and long-term healing. In contrast, the PG/Van/CB and PG/Van/B/CB groups showed that the CB materials uniformly filled the defect area, while the PG hydrogel had largely degraded. New bone tissue effectively filled the gaps between the CB and the defect margins, demonstrating fusion between the bone graft materials and the newly formed bone tissue, with partial trabecular formation and mineralization within the materials. However, the newly formed bone tissue in the PG/Van/CB group was sparse and loose, whereas in the PG/Van/B/CB group, it was abundant and dense. In summary, the PG/Van/B/CB group demonstrates the most effective bone repair, showing potential for future clinical application. One of the advantages of bioadhesive systems is their ability to serve as effective vehicles for delivering functional additives. In the future, rhBMP-2 and Van may be modified or combined with other additives for tailored therapies.

## 4 Conclusion

In summary, this study introduces a method of fabricating bone/bioadhesive grafts delivery system based on through a bidirectionally enhanced approach. Additionally, the hydrogel enables the injectable delivery of autologous, allograft, and artificial bone while exhibiting shape adaptability and osteoadhesion, showcasing great potential for bone graft material delivery. Meanwhile, after loading the bone granules, the system possesses excellent sealing properties, thereby enhancing its overall performance. The hemostasis of femoral artery hemostasis in SD rat and femoral condylar defects in New Zealand rabbit showed PG/CB had superior hemostatic sealing performance and efficiency, possessing the potential to be used as bone hemostatic materials. Another function of this delivery system is that it can easily load different functional additives. By loading Van, the system can effectively reduce the risk of infection following surgery for open bone defects. Moreover, the bone/bioadhesive graft delivery system loaded with rhBMP-2 enhances the osteoinductive activity of CB, thereby promoting osteogenesis in the subcutaneous tissue of SD rats and facilitating cranial bone regeneration.

Overall, the PG/Van/B/CB bone/bioadhesive grafts delivery system can be characterized as a biomaterial with transformative capabilities. This system can function as a hemostatic material during surgery, an antibacterial and antioxidant material in the early postoperative, and an osteogenic material in the long-term postoperative. In the future, rhBMP-2 and Van may be modified or combined with other additives for tailored therapies. Therefore, the bone/bioadhesive grafts delivery system represents a promising option for preventing infection in open bone defects.

However, during clinical application, attention should be paid to the size of the bone grafts, and the thrombus risk management system should be optimized to prevent bone grafts from entering the circulatory system and forming embolisms. Meanwhile, it is noteworthy that the PB/CB still exhibits significant swelling in this study, although swelling can promote liquid sealing to achieve hemostasis and nutrient exchange. Considering that this system will be applied to different bone defect sites, it is necessary to evaluate the influence of swelling behavior on different applications in future studies to minimize the impact on sensitive tissues, such as nerves and the brain. Meanwhile, an infectious bone regeneration model is not selected for this experiment, and subsequent research will need to explore the systems’ efficacy in the standardized infectious bone defect model. Additionally, given the physiological differences among different species, it is essential to study the safety of the bone grafts/bioadhesives in other animal models, especially large animal, before considering clinical translation applications. Moreover, designing systems with the ability of underwater adhesion is very important for applications, especially in minimal invasive surgery in the highly humid environment.

## CRediT authorship contribution statement

**Qingzu Liu:** Writing – original draft, Validation, Software, Methodology, Investigation, Funding acquisition, Data curation. **Bin Zhu:** Writing – review & editing, Software, Methodology, Formal analysis, Conceptualization. **Huikai Yang:** Investigation, Data curation. **Chongyang Liu:** Investigation, Formal analysis. **Yurong Chen:** Writing – review & editing, Visualization. **Xiaoyu Wu:** Writing – review & editing, Investigation. **Wangling Duan:** Software. **Luyao Feng:** Writing – review & editing. **Binhui Wang:** Investigation. **Liang Shao:** Data curation. **Jianpeng Gao:** Data curation. **Yazhong Bu:** Project administration, Investigation, Funding acquisition, Data curation. **Hongjian Liu:** Resources, Project administration, Conceptualization. **Keya Mao:** Writing – review & editing, Supervision, Resources, Project administration, Conceptualization. **Jianheng Liu:** Writing – review & editing, Supervision, Resources, Project administration, Funding acquisition, Conceptualization.

## Ethics approval and consent to participate

All animal studies were performed in accordance with Xi'an Jiaotong University Ethics Committee on the use of Animals in Research and Teaching with the number XJTUAE2024-2037. SD rats (weight of 200–220 g) and New Zealand rabbits (weight of 2.0-2.5 kg) were used in animal studies. Animals were housed in plastic cages at a constant temperature (26 °C) and relative humidity (70%) with a fixed 12 h light/dark cycle and free access to food and water. Animals were randomly assigned to different groups in the study.

## Declaration of competing interest

The authors declare that they have no known competing financial interests or personal relationships that could have appeared to influence the work reported in this paper.
