# Supplementary material for: Engineering injectable bone/bioadhesive grafts delivery system with self-healing properties for bone regeneration
Source: Bioact Mater. 2025 Aug 11;54:47–70. doi: 10.1016/j.bioactmat.2025.07.049 (PMC12358664; doi:10.1016/j.bioactmat.2025.07.049)
Supplement: Multimedia component 1 [file mmc1.docx]

Supplementary Materials

**Engineering injectable bone/bioadhesive grafts delivery system with self-healing properties for bone regeneration**

**
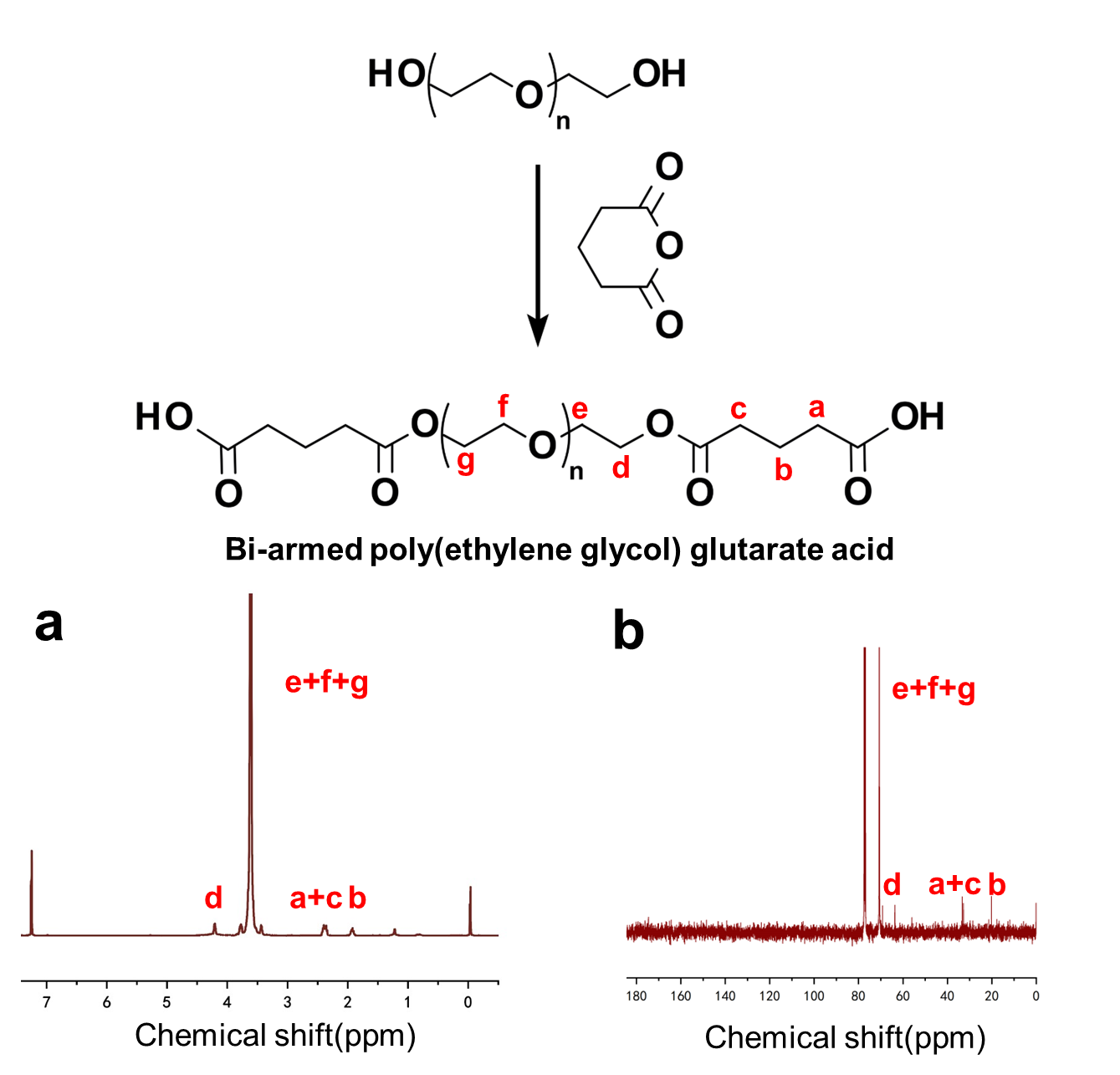
**

**Figure S1.** Synthetic route of the Bi-armed poly (ethylene glycol) glutarate acid and its (a) ^1^H NMR and (b) ^13^C NMR spectra in CDCl3.


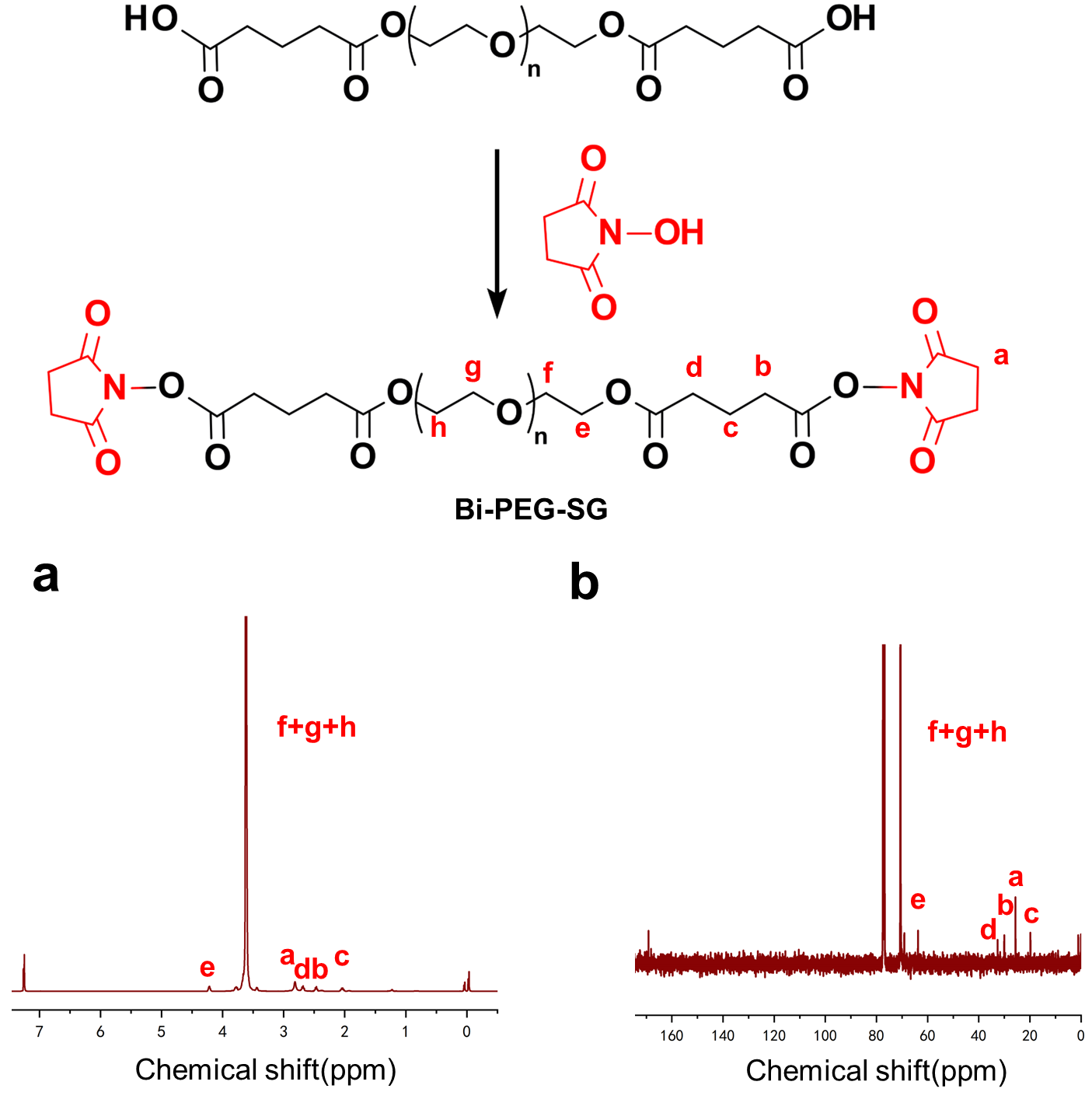


**Figure S2.** Synthetic route of the Bi-PEG-SG and its (a) ^1^H NMR and (b) ^13^C NMR spectra in CDCl_3_.


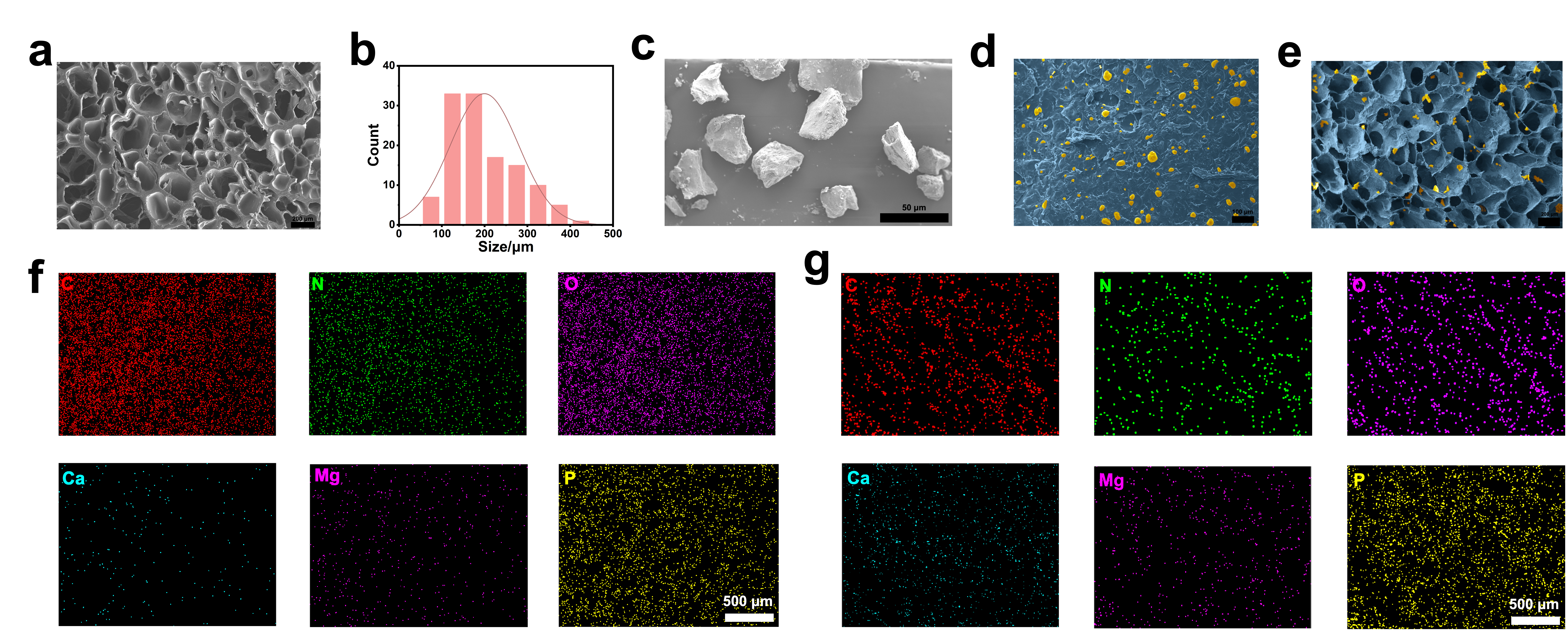


**Figure S3.** a) SEM image of PG hydrogel. b) The pore size distribution of PG hydrogel. c) Surface morphology calf bone. d) Surface of bone/bioadhesive grafts delivery system. e) Cross-section of bone/bioadhesive grafts delivery system. f) Surface EDS of bone/bioadhesive grafts delivery system. g) Cross-section EDS of bone/bioadhesive grafts delivery system.


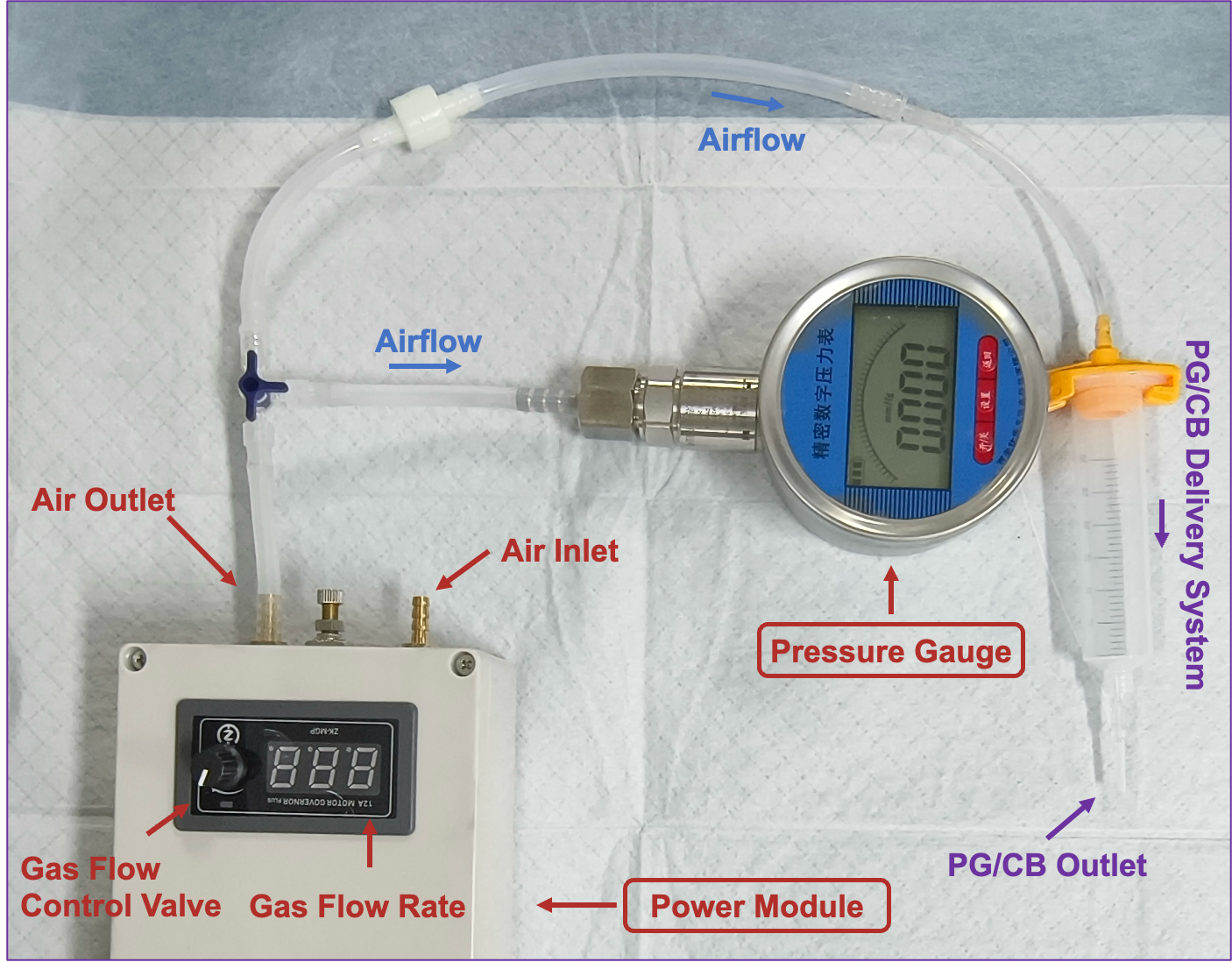


**Figure S4.** Schematic diagram of pneumatic injection system


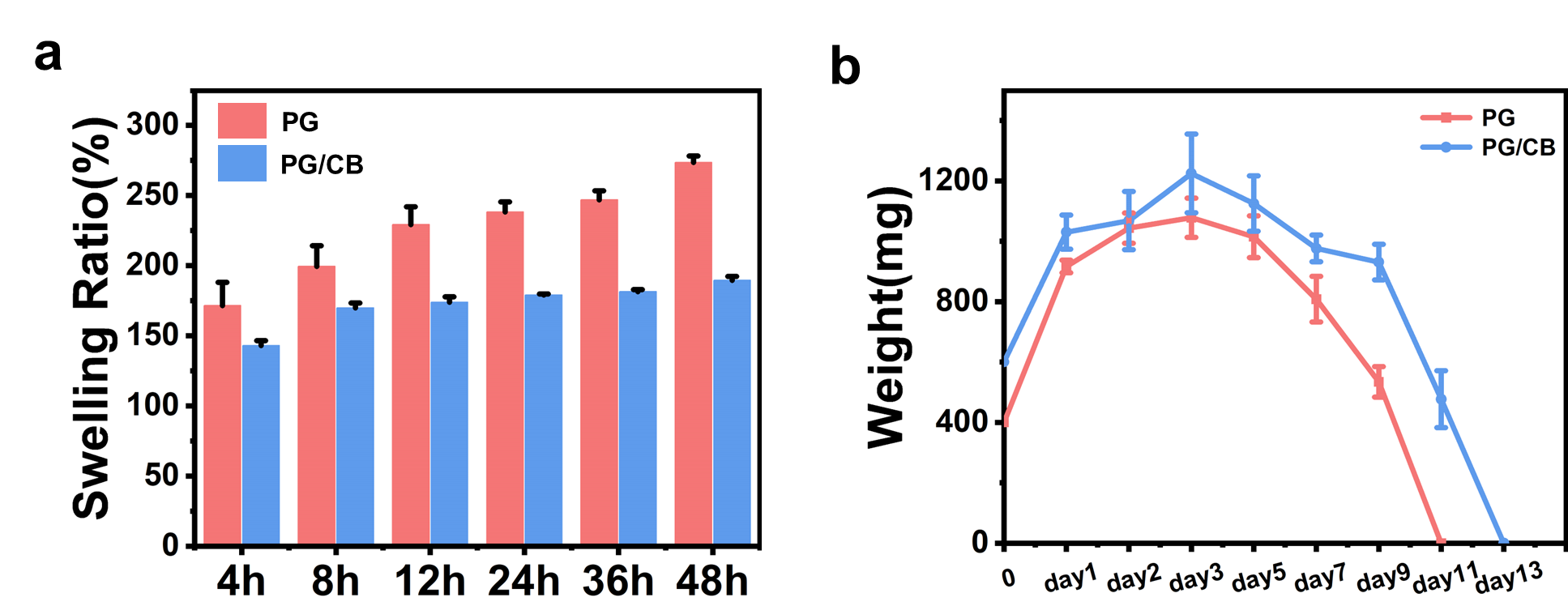


**Figure S5.** Swelling ratio of PG and PG/CB (a), In vitro degradation curves of PG and PG/CB (b).


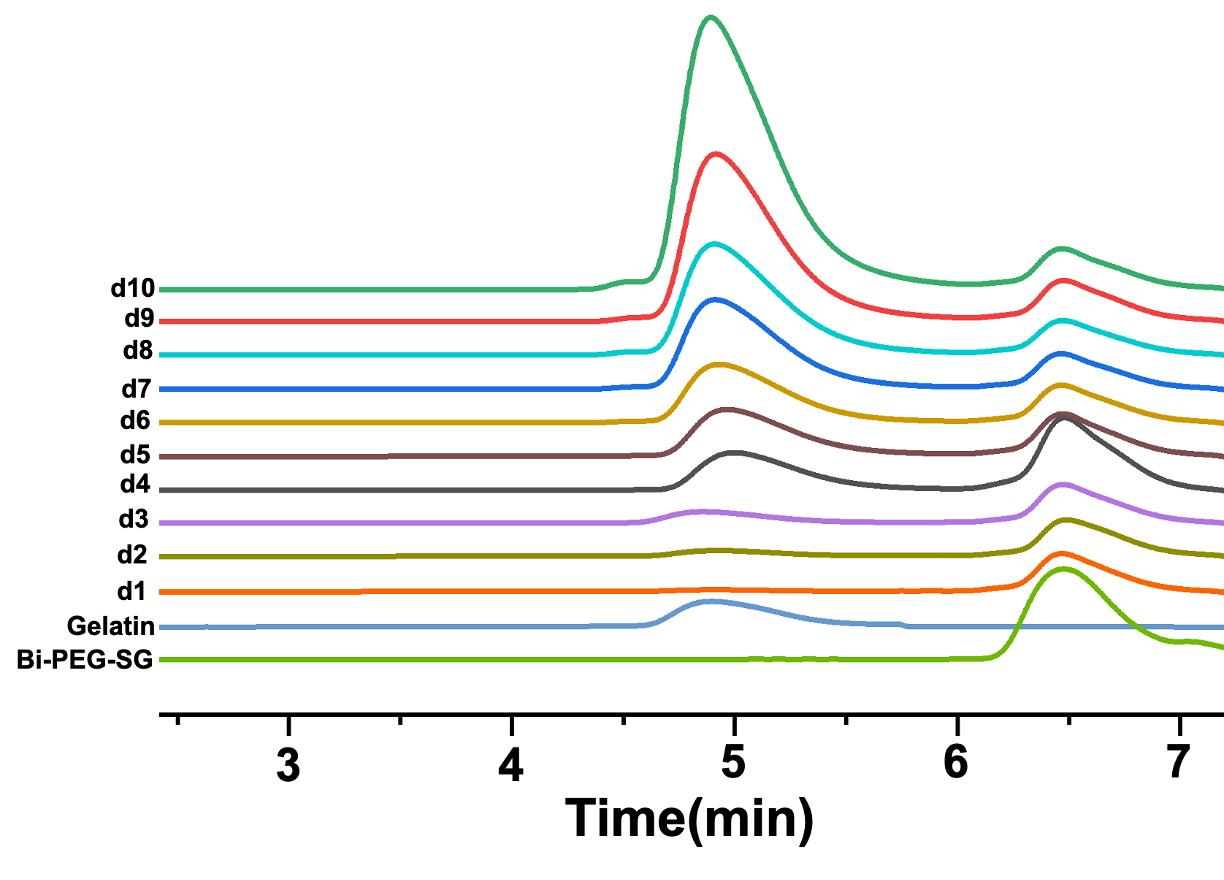


**Figure S6.** Advanced Polymer Chromatography Plot of Degradation Products at Different Times.

Table S1: Molecular weight of degradation products with different degradation times

| Degradation time | - | d1 | d2 | d3 | d4 | d5 | d6 | d7 | d8 | d9 | 10 |
| --- | --- | --- | --- | --- | --- | --- | --- | --- | --- | --- | --- |
| PEG-Mn | 6235 | - | - | - | - | - | - | - | - | - | - |
| Gelatin-Mn | 76466 | - | - | - | - | - | - | - | - | - | - |
| Peak1-Mn | - | 90220 | 83656 | 82493 | 65317 | 68784 | 72493 | 74853 | 75245 | 74445 | 77294 |
| Peak1-DPI | - | 1.154 | 1.181 | 1.128 | 1.159 | 1.153 | 1.14 | 1.138 | 1.147 | 1.18 | 1.149 |
| Peak2-Mn | - | 5970 | 5743 | 6737 | 5823 | 5881 | 5655 | 5691 | 5380 | 5818 | 5775 |
| Peak2-DPI | - | 1.47 | 1.577 | 1.138 | 1.537 | 1.51 | 1.448 | 1.577 | 1.469 | 1.522 | 1.514 |


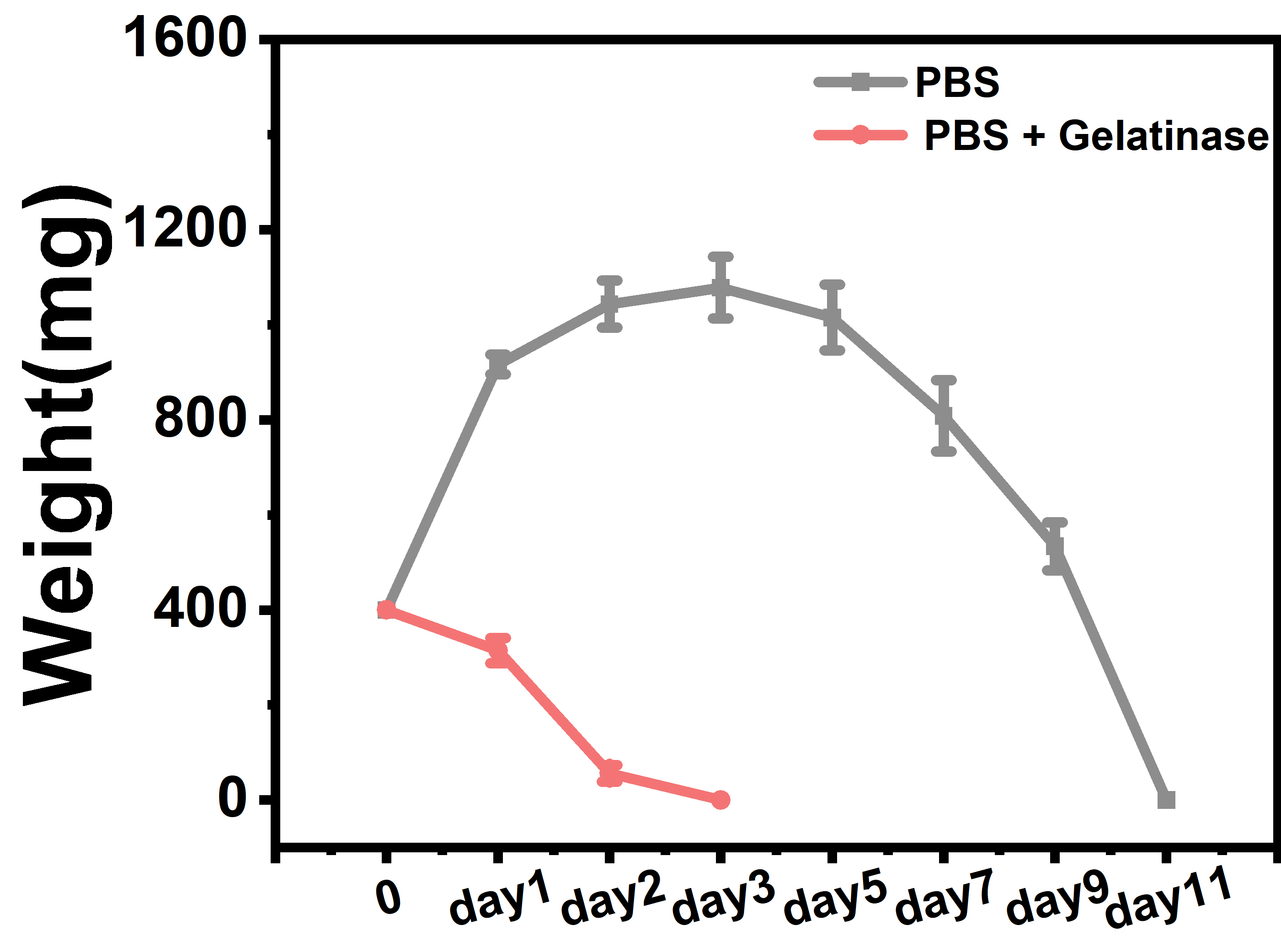


**Figure S7.** PG hydrogel in blank PBS and PBS + Gelatinase (2 U/mL) at 37 ℃.


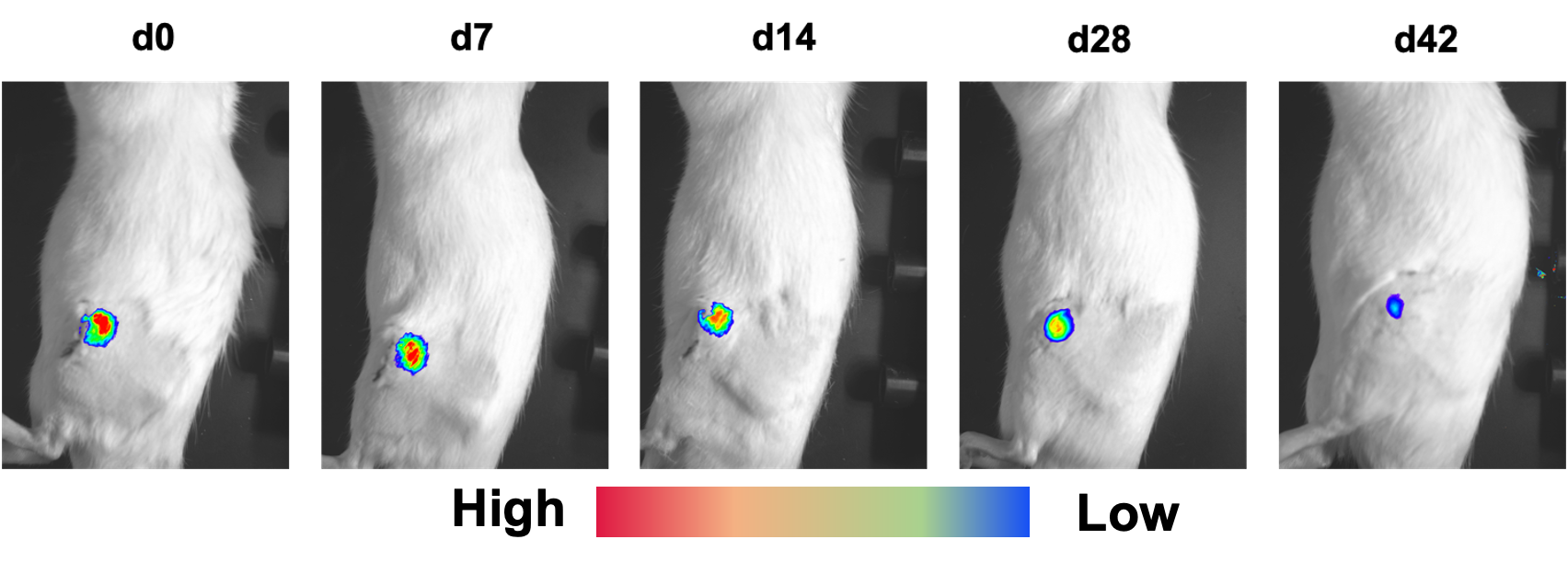


**Figure S8.** The degradation of PG hydrogel in femoral condylar defect imaging in *vivo.*


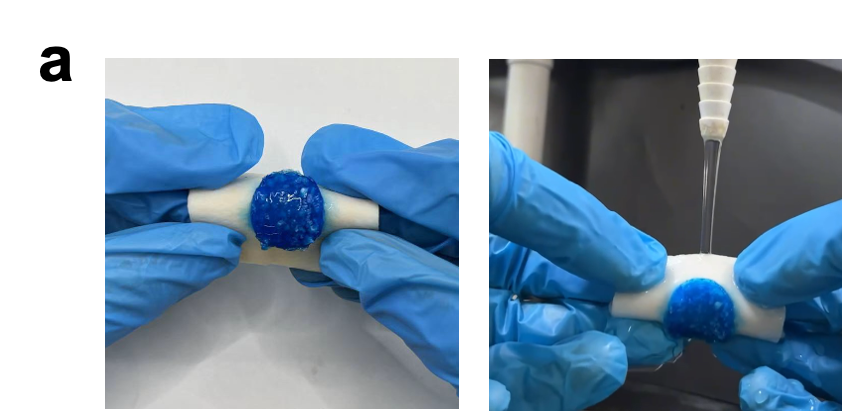


**Figure S9.** Presentation the adhesion of PG/CB delivery system in porcine skin.


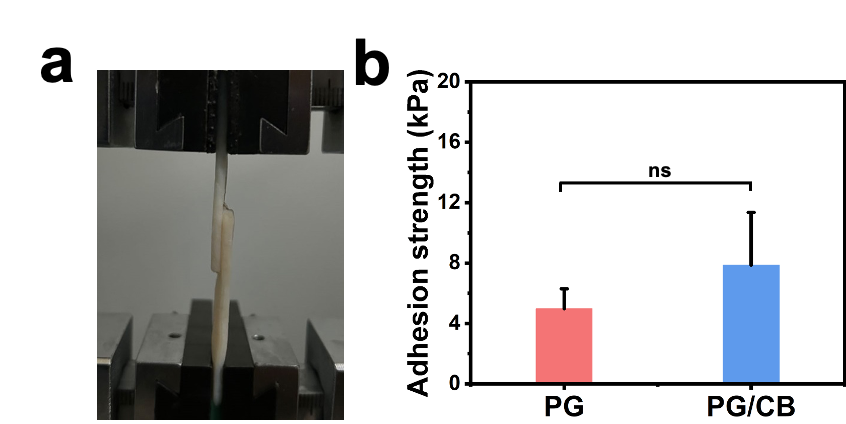


**Figure S10.** Presentation the adhesion strength of PG/CB delivery system in porcine skin.

Table S2 q-PCR primer sequences

| primer | sequences |
| --- | --- |
| OPN - forward | 5′ - GCAGGACTAACTACGACCATGAG - 3′ |
| OPN - reverse | 5′ - TTCTTCAGAGGACACAGCATTCT - 3′ |
| OCN - forward | 5′ - GGGCAATAAGGTAGTGAACAGACT - 3′ |
| OCN - reverse | 5′ - CTGGTCTGATAGCTCGTCACAAG - 3′, |
| RUNX2 - forward | 5′ - GGCCACTTACCACAGAGCTATTA - 3′ |
| RUNX2 - reverse | 5′ - AGGCGATCAGAGAACAAACTAGG - 3′ |
| COL1 - forward | 5′ - GCCGCAAAGAGTCTACATGTCTA - 3′ |
| COL1 - reverse | 5′ - AGTCCAGTTCTTCATTGCATTGC - 3′ |
| β-actin - forward | 5′-CGTTGACATCCGTAAAGACCTCTA-3′ |
| β-actin - reverse | 5′- CATCGTACTCCTGCTTGCTGATC -3′ |


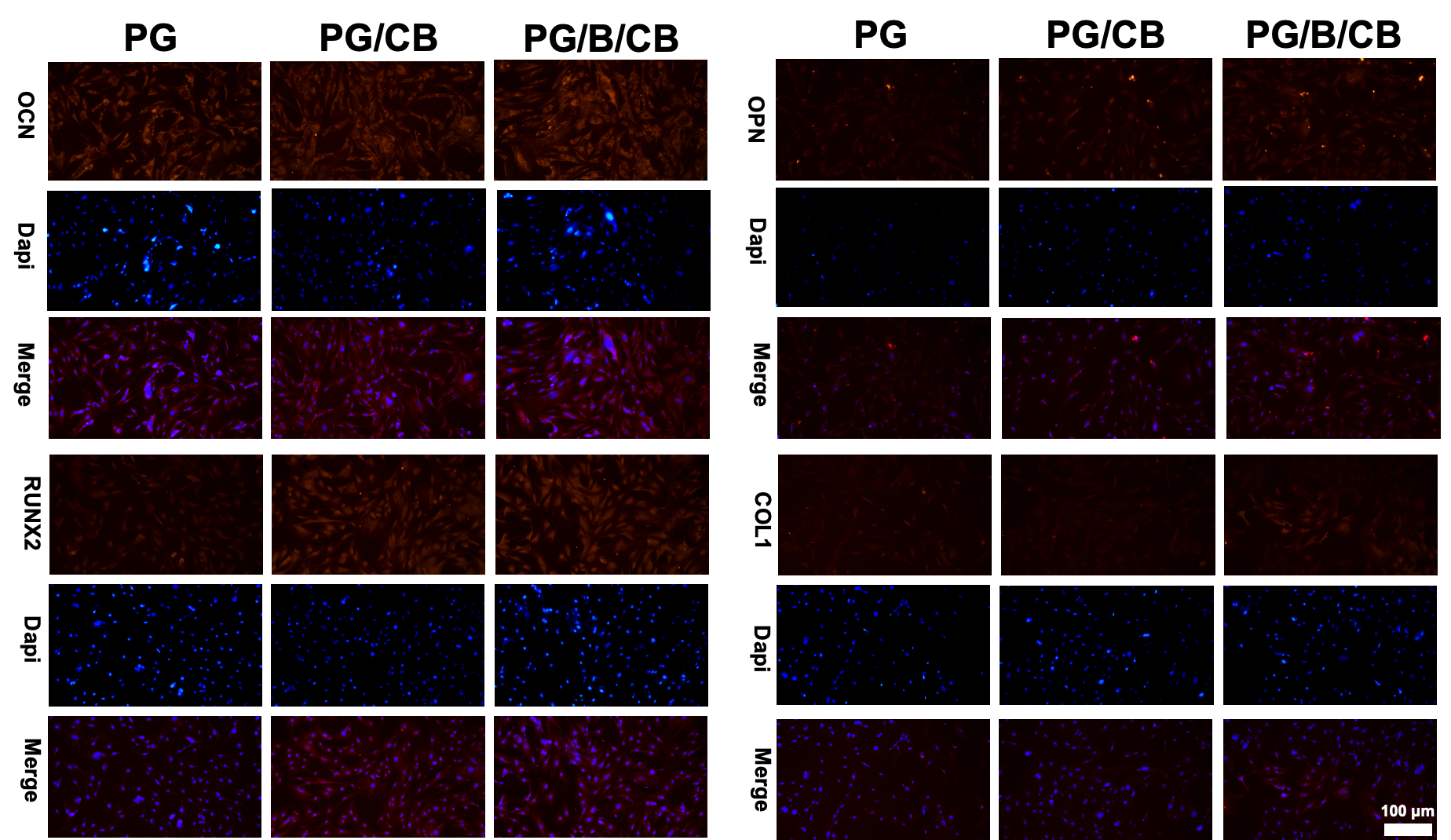


**Figure S11.** Immunofluorescence of osteogenic proteins (OPN, OCN, RunX2 and COL1), (Scale bar = 100 μm).


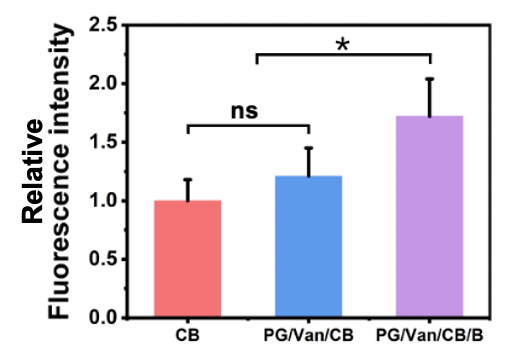


**Figure S12.** Relative fluorescence intensity of CD31 at 4 weeks.


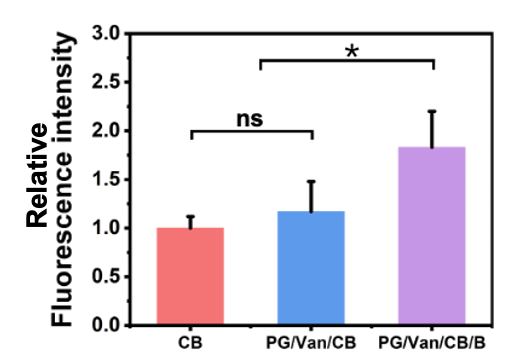


**Figure S13.** Relative fluorescence intensity of HIF1 at 4 weeks.
